# Innovating spinal muscular atrophy models in the therapeutic era

**DOI:** 10.1242/dmm.050352

**Published:** 2023-10-03

**Authors:** Ilaria Signoria, W. Ludo van der Pol, Ewout J. N. Groen

**Affiliations:** Department of Neurology and Neurosurgery, UMC Utrecht Brain Center, University Medical Center Utrecht, 3584 CX Utrecht, the Netherlands

**Keywords:** Gene therapy, Motor neuron disease, Spinal muscular atrophy, Neuromuscular disease

## Abstract

Spinal muscular atrophy (SMA) is a severe, monogenetic, neuromuscular disease. A thorough understanding of its genetic cause and the availability of robust models has led to the development and approval of three gene-targeting therapies. This is a unique and exciting development for the field of neuromuscular diseases, many of which remain untreatable. The development of therapies for SMA not only opens the door to future therapeutic possibilities for other genetic neuromuscular diseases, but also informs us about the limitations of such treatments. For example, treatment response varies widely and, for many patients, significant disability remains. Currently available SMA models best recapitulate the severe types of SMA, and these models are genetically and phenotypically more homogeneous than patients. Furthermore, treating patients is leading to a shift in phenotypes with increased variability in SMA clinical presentation. Therefore, there is a need to generate model systems that better reflect these developments. Here, we will first discuss current animal models of SMA and their limitations. Next, we will discuss the characteristics required to future-proof models to assist the field in the development of additional, novel therapies for SMA.

## Teaser

**Summary:** Recently approved gene-targeting therapies for spinal muscular atrophy are leading to milder and more variable phenotypes in patients. Here, we discuss requirements for next-generation disease models to incorporate these therapeutic developments.

## Introduction

Spinal muscular atrophy (SMA) is a severe, monogenetic neuromuscular disease characterised by progressive muscle weakness caused by loss or dysfunction of motor neurons (Mercuri et al., 2022). The severity of SMA varies significantly, and it is traditionally classified based on motor milestones achieved and the age of onset (Groen et al., 2018a; Wadman et al., 2017). The most severe type, SMA type 1, is also the most common type of the disease and, when untreated, leads to death around one year after birth, before any motor milestones are achieved. SMA type 4, in contrast, is the mildest and also rarest type of the disease, characterised by adult onset and affected individuals retaining the ability to walk independently (Piepers et al., 2008). Between these extremes, type 2 and type 3 make up a large group of patients that, in addition to limited motor function, can develop any of a range of complications including scoliosis, bulbar symptoms, increased fatiguability and respiratory complications (Wadman et al., 2017). Additionally, it is increasingly recognised that symptoms outside the central nervous system (CNS), including dysfunction of the kidney, liver, heart and bone, may affect some patients to varying degrees (Hamilton and Gillingwater, 2013; Yeo and Darras, 2020). In general, although the life expectancies of patients with SMA types 2, 3 and 4 are close to normal (Wijngaarde et al., 2020), muscle weakness and complications caused by SMA can significantly deteriorate the quality of life (Wohnrade et al., 2023). Without exception, SMA is always progressive when untreated.

**Fig. 1.**
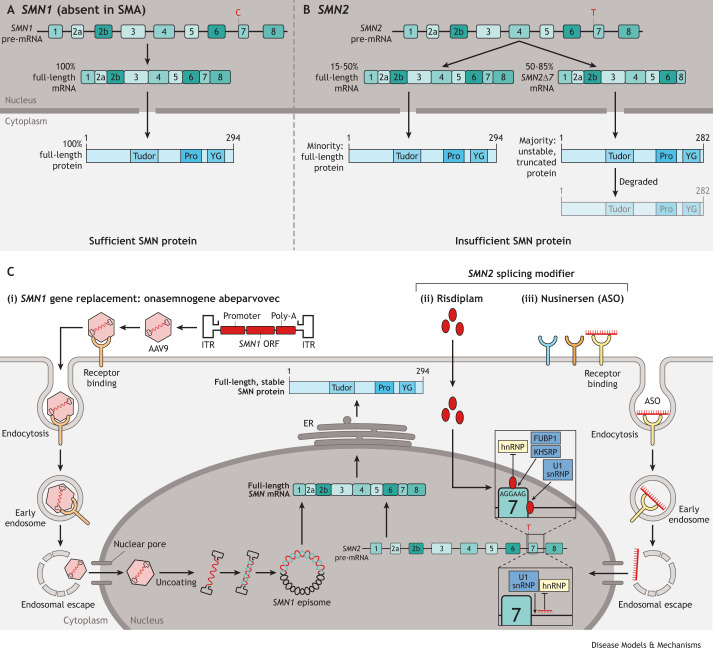
**Overview of the genetics of SMA and the mechanisms of SMN-targeting treatments.** (A) In unaffected individuals, at least one copy of *SMN1* is present in the highly variable segmental duplication on chromosome 5q13. *SMN1* is transcribed into full-length *SMN* pre-mRNA, which is spliced into a mature mRNA (*FL-SMN*) that contains all nine exons of the coding part of the gene. *FL-SMN* is translated into the full-length, 294 amino acid SMN protein that contains multiple domains related to SMN function and localisation. Although SMN protein expression varies between cell types and developmental time points, the presence of a functional copy of *SMN1* always leads to the production of physiologically sufficient SMN protein levels. (B) In patients with SMA, *SMN1* function is lost through homozygous deletion or loss-of-function mutations. Patients therefore depend on the paralogous *SMN2* for SMN protein production. However, a single nucleotide change from cytosine to thymine leads to the exclusion of exon 7 in the majority of *SMN2*-derived RNA transcripts (*SMN2-*Δ*7*). This alternative splicing leads to the translation of a truncated, 282 amino acid SMN protein from the majority of *SMN2*-derived transcripts. This is an unstable protein that is quickly degraded. Although a high *SMN2* copy number leads to higher SMN protein levels, in the absence of functional *SMN1* or protective, rare, genetic modifier variants, *SMN2*-derived protein levels are always physiologically insufficient and eventually lead to pathology. (C) Three currently approved drugs each target SMA on a genetic level and increase SMN protein levels. (i) With onasemnogene abeparvovec-xioi (Zolgensma) treatment, adeno-associated virus 9 (AAV9) containing the *SMN1* open reading frame (ORF) under the control of the cytomegalovirus enhancer/chicken-β-actin-hybrid promoter and with a poly-A tail is distributed throughout the body via a one-off intravenous injection. After entering the cell by endocytosis, the virion escapes from the endosome, after which it can enter the nucleus through nuclear pores. After uncoating, double-stranded DNA is formed, and the transgene is expressed from episomes. Although viral genome copy number varies extensively between cell types, the presence of nuclear episomes is thought to lead to persistent, stable expression of the full-length SMN protein, especially in non-dividing cells. (ii) With risdiplam (Evrysdi) treatment, the small molecule enters cells by passive diffusion, possible due to its high passive permeability. Currently, there is no general consensus on its mechanism of action, and all mechanistic studies so far have been performed using risdiplam analogues. It is likely that SMN-C3, one such analogue, interacts with an AG-rich motif in exon 7, leading to the displacement of heterogeneous nuclear ribonucleoproteins (hnRNPs) and to the recruitment of stimulatory factors, including FUBP1 and KHSRP (Wang et al., 2018). In addition, it has been shown that SMN-C5, another analogue, interacts with the 5′ splicing site of exon 7, promoting the recruitment of U1 small nuclear ribonucleoprotein (snRNP) (Campagne et al., 2019). Regardless of the exact mode of action, the final result is the increased inclusion of exon 7 and subsequent increased SMN protein levels. (iii) With nusinersen (Spinraza) treatment, antisense oligonucleotides (ASOs) are injected intrathecally. Although detailed studies on nusinersen-specific cellular uptake mechanisms have not yet been published, previous studies on other ASOs suggest that uptake mostly happens through clathrin or caveolin-dependent endocytosis, likely facilitated by several cell surface proteins such as integrins, G protein-coupled and scavenger receptors (Crooke et al., 2017). Once internalised, ASOs can be trafficked from early endosomes to late endosomes and to lysosomes. Importantly, to reach their target mRNAs, ASOs must escape from the endosomal pathway. Once nusinersen enters the nucleus, splicing correction of *SMN2* pre-mRNA happens through binding of nusinersen to the intronic splicing silencer N1 region upstream of *SMN2* exon 7. Splicing and splice correction involve many regulatory sequences and factors. Briefly, nusinersen binding causes structural rearrangements that block the hnRNP-binding site and increase accessibility of the TIA1-binding site, which leads to the recruitment of the U1 small nuclear ribonucleoprotein (snRNP) to the 5′ splice site of exon 7. This process – similar to that of risdiplam - results in the enhanced inclusion of exon 7 and increased SMN protein levels. AAV, adeno-associated virus; ASO, antisense oligonucleotide; ER, endoplasmic reticulum; FUBP1, far upstream element-binding protein 1; hnRNPs, heterogeneous nuclear ribonucleoproteins; ITR, inverted terminal repeat; KHSRP, KH-type splicing regulatory protein; ORF, open reading frame; Pro, proline-rich region; SMN, survival of motor neuron; TIA1, cytotoxic granule associated RNA binding protein TIA1; U1 snRNP, U1 small nuclear ribonucleoprotein; YG, YG-box domain.

SMA is caused by deleterious variants in the survival of motor neuron 1 (*SMN1*) gene (Lefebvre et al., 1995). The paralogous, but less functional, gene *SMN2* is the main modifier of SMA severity. A cytosine-to-thymine variant in exon 7 of *SMN2* causes the majority of transcripts to be alternatively spliced, leading to lower levels of the functional, full-length SMN protein (Fig. 1A,B) (Lorson et al., 1999; Monani et al., 1999). However, because the *SMN* locus on chromosome 5 is complex and unstable, significant copy number variation occurs, with *SMN2* copy numbers ranging from one to eight copies (Wirth et al., 2020). In general, a higher *SMN2* copy number leads to higher SMN protein levels and therefore a relatively milder phenotype (Wadman et al., 2020). This correlation is especially clear at both ends of the SMA severity spectrum: most patients with SMA type 1 have two *SMN2* copies, whereas all patients with SMA type 4 have at least four *SMN2* copies. However, the correlation between *SMN2* copy number and disease severity for SMA types 2 and 3 is less clear, and the copy number for these subtypes usually ranges between two and four (Calucho et al., 2018; Wadman et al., 2020). In addition to *SMN2* copy number variation, other genetic modifiers of SMA have also been described (Hosseinibarkooie et al., 2016; Oprea et al., 2008; Riessland et al., 2017), but up to 40% of variation in disease severity remains genetically unexplained. In a large group of patients with three or four copies of *SMN2*, prognosis remains difficult to provide based on genotype alone.

Treatment for patients with the most severe types of SMA used to be mostly palliative, and for patients with relatively less severe types, it was mostly supportive (e.g. physical therapy, scoliosis surgery and feeding support). Excitingly, a thorough understanding of the genetic cause of SMA recently led to the approval of three gene-targeting, disease-modifying therapies (Fig. 1C) (Chaytow et al., 2021), representing a milestone in the treatment of neuromuscular diseases. Although treatment response varies from significant clinical improvement to initial stabilisation, the majority of patients with SMA appear to benefit from these therapies.

The success story of SMA therapies was built, in large part, on the availability of multiple, robust disease models ranging from cell-based models to small animals (Table 1) – such as *Caenorhabditis elegans* (Briese et al., 2008; Doyle et al., 2020; Sleigh et al., 2011a), *Drosophila* (Chan et al., 2003; Chang et al., 2008; Spring et al., 2019) and zebrafish (Boon et al., 2009; McWhorter et al., 2003; Tay et al., 2021) – and many mouse models (Table 2) (Bowerman et al., 2009, 2012a; Hsieh-Li et al., 2000; Le et al., 2005; Monani et al., 2000). These models allowed key aspects of SMA pathology to be characterised and gene-targeting therapies to be investigated, tested and optimised *in vitro* and *in vivo.* Preclinical studies performed in these models generated invaluable data on the efficacy, distribution and other key requirements of gene-targeting therapies (Foust et al., 2009; Hua et al., 2011; Zhao et al., 2021). Importantly, these findings proved to be highly translatable to patients, leading to the relatively efficient and rapid clinical development of gene-targeting therapies for SMA.

**Table 1.**
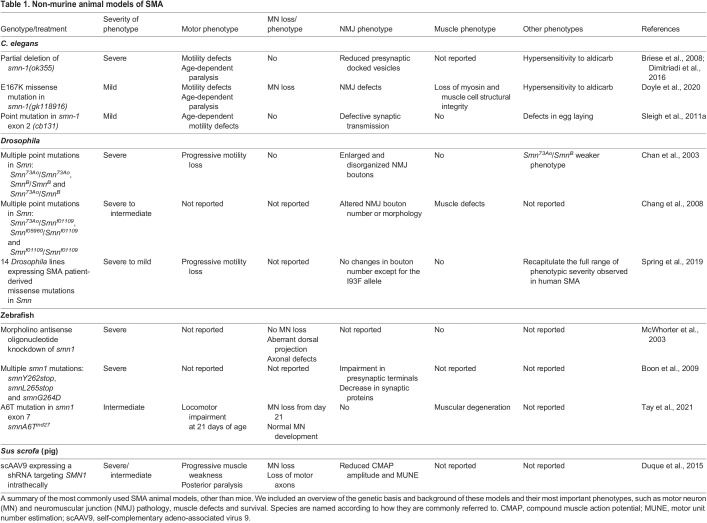
Non-murine animal models of SMA

**Table 2.**
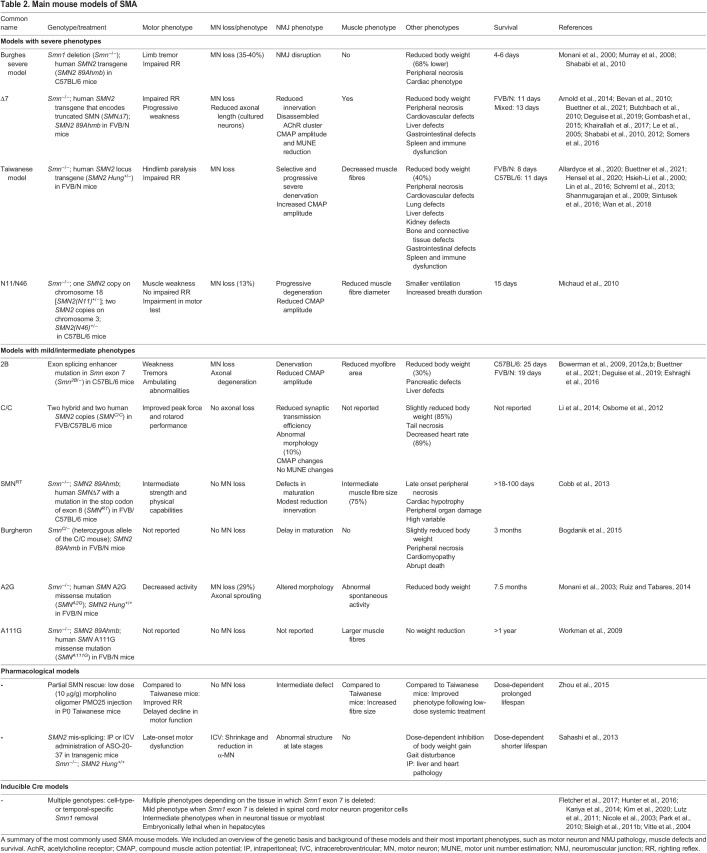
Main mouse models of SMA

Now that three gene-targeting therapies are used extensively in clinics around the world, follow-up data of treated SMA patients are becoming available for large, real-world patient cohorts (Antonaci et al., 2023; Scheijmans et al., 2022). These studies highlight some important limitations of current therapies with regards to variability in therapy response, and illustrate that the cause of variability in disease severity and treatment outcomes remains incompletely understood. Existing SMA animal models present a disease that is genetically and phenotypically much more homogeneous than in patients, and thus their use to study the underlying causes of this variability may be limited. In addition, current gene-targeting therapies lead to a shift towards milder phenotypes in patients, highlighting the need to develop models that also recapitulate less severe disease. Now is therefore an important time to reflect on the use of current disease models in SMA research and discuss further possibilities to develop and innovate existing and new models, focusing on key features of SMA in the therapeutic era. This will enable the optimisation of gene-targeting therapies to maximise their benefits and the development of novel therapies that combat the remaining symptoms.

## SMN-targeting treatments change the clinical landscape of SMA

The approval of SMN-targeting therapies has significantly changed disease course and outcomes of SMA. Although drug approval and the start of widespread use of therapies in real-world settings is relatively recent, the majority of patients who started treatment after symptom onset experienced a degree of benefit from receiving these therapies (Antonaci et al., 2023). This ranges from significant clinical improvement in young children who started treatment early to stabilisation of the disease in the majority of older children and adults. The biggest treatment effects have been observed in children for whom treatment was started as early as possible and at a time when motor function was still well-maintained.

Each treatment uses a different approach to increase SMN expression (Fig. 1C): (1) nusinersen (Spinraza) is an antisense oligonucleotide (ASO; see Glossary, Box 1) *SMN2*-splicing modifier (Finkel et al., 2017; Hua et al., 2008; Mercuri et al., 2018); (2) onasemnogene abeparvovec-xioi (Zolgensma) is an adenoviral vector-based *SMN1* gene-replacement therapy (Mendell et al., 2017; Valori et al., 2010); and (3) risdiplam (Evrysdi) is a small-molecule *SMN2*-splicing modifier (Baranello et al., 2021; Naryshkin et al., 2014). As these drugs require different delivery routes – intrathecal injection, one-off intravenous infusion and oral delivery, respectively – the choice of treatment for patients is often pragmatic and based on, for example, age, weight or the possibility to access the spinal column for intrathecal injection. As a consequence, the different characteristics and delivery routes lead to CNS-specific, partially systemic or systemic increases of SMN. This currently complicates interpretation of the clinical progression of SMA after the start of treatment in symptomatic patients. Treatment efficacy may appear more limited for some drugs than for others as more severely affected patients are more likely to be treated with a certain drug. For example, adult patients with severe scoliosis may not be able to receive nusinersen owing to limited access to the spinal column for intrathecal injection, whereas less severely affected patients who receive nusinersen may be more likely to respond to the treatment (Erdos and Wild, 2022). Next, as some of the therapies lead to CNS-specific or partially systemic increases of SMN levels in specific groups of patients (Ramos et al., 2019; Thomsen et al., 2021) systemic phenotypes of SMA that were previously considered subclinical may become more pronounced in groups of patients that receive those treatments. Additionally, the wide range in severity and disease duration before patients start treatment complicates direct comparisons between treatments and patient groups. These points illustrate the challenges in discussing prognosis and expected treatment benefit with patients, as many features of SMA treatment remain uncertain. Long-term follow-up with patients receiving treatment is therefore of the utmost priority.

**Box 1. Glossary**

**Adeno-associated viruses (AAVs):** small, single-stranded DNA viruses that infect humans and some other primate species. As AAVs cause low-level immune responses, they are considered more safe than lentiviruses for, for example, gene therapy.

**Antisense oligonucleotides (ASO):** short, synthetic, single-stranded oligodeoxynucleotides that can form complementary pairs with a target mRNA. They can be designed to modulate gene expression, splicing, RNA stability or RNA post-transcriptional modifications.

**Axonal defect:** defects in the portion of a neuron that carries impulses away from the cell body, such as structural defects, connectivity defects, impaired development or degeneration.

**Hybrid genes:** although highly similar, 16 paralagous sequence variants (PSVs) have been described that differ between *SMN1* and *SMN2*. *SMN2* copies that have a combination of *SMN1*- and *SMN2*-specific PSVs are referred to as hybrid genes.

**Maternal gene contribution:** the effects of a maternal gene when its products (RNA or protein) are produced or deposited in the oocyte or are present in the fertilized egg or embryo before the expression of zygotic genes is initiated.

**Modifier genes:** genes in which variants can modify the phenotypic outcome of the primary disease-causing gene.

**Motor neuron (MN):** α-motor neurons of the ventral spinal cord, which are the main cell type affected in SMA. As these motor neurons directly innervate muscles throughout the body, degeneration leads to progressive weakness and muscle atrophy.

**Neuromuscular junction (NMJ):** the chemical synapse between a motor neuron and a muscle fibre.

**Righting reflex (RR):** the reflex resulting in the body tending to regain its former body position when it is in the supine position.

***SMN-AS1*:** a long non-coding RNA that arises from the antisense strand of *SMN* and regulates *SMN* mRNA levels.

Excitingly, the availability of multiple treatments and the recognition that early start of treatment leads to significantly better outcomes is now leading to the inclusion of SMA in newborn screening programmes around the world (Dangouloff et al., 2021). Early genetic diagnosis of SMA (generally within two weeks after birth) allows the start of treatment before symptom onset in most patients. Recent phase III studies on onasemnogene abeparvovec in presymptomatic children with two or three copies of *SMN2* showed that, although varying degrees of delayed motor development remained, many children developed in line with or close to the normal developmental window for newborns (Strauss et al., 2022a,b). For this specific group of patients, it is still unclear if and to what extent they will develop features of SMA in later life, and what will be the best approach to monitor putative disease progression in the future. In addition, and especially for children with two *SMN2* copies, it is unclear to what extent they will develop new, unexpected symptoms in the long term that may be different from those that we have previously associated with SMA. For example, in Pompe disease – a rare metabolic disease leading to progressive muscle weakness – enzyme replacement therapy has been very effective in improving development and survival, but can unexpectedly lead to progressive white-matter abnormalities later in life (Ebbink et al., 2018). As for symptomatic SMA patients, careful long-term monitoring in real-world populations will be vital to better inform patients and clinicians for expected prognosis of disease severity and long-term treatment effects.

In summary, despite its monogenetic origin, SMA is a complex disease. SMA prognosis was never straightforward because of an incomplete correlation between genotype and disease severity. The approval of different types of therapies administered at various stages of the disease further increases the uncertainty around disease severity and therapeutic outcomes for clinicians, and patients with SMA and their families.

## An overview of current disease models

To study SMA pathogenesis and support drug development, a wide range of SMA model systems are available. A detailed discussion of all models would go beyond the scope of this Review, but an overview of the most commonly used models has been included in Tables 1 and 2.

Humans and several closely related Homininae species are the only organisms with *SMN* duplication, and the *SMN2* gene is unique to humans (Rochette et al., 2001). All other organisms carry one SMN gene only and therefore cannot develop SMA, as a complete loss of *SMN* is embryonically lethal (Schrank et al., 1997). The ability to model SMA therefore depends on the use of a variety of genetic approaches, including the use of *SMN2* transgenes, partial-loss-of-function mutations or knockdown of *SMN1*, and hybrid genes (Box 1). However, the SMN protein is evolutionarily and functionally well conserved (Osman et al., 2019), which means that overlapping mechanisms and phenotypes are present in many SMA models. The most common and widely investigated phenotypes in SMA are related to defects in the neuromuscular system, such as loss of motor neurons (Box 1), axonal defects (Box 1), neuromuscular junction (NMJ; Box 1) dysfunction or muscle-specific pathology. The specifics of these phenotypes, their severity and how they have been studied depend on the possibilities and limitations of the model that is used.

Many species and approaches have been used to study SMA and range from small invertebrate models to large animal models, such as pigs. The possibilities for genetic manipulation to induce SMA phenotypes differ between models and species. For instance, *Smn* knockout in *Drosophila*, *C. elegans* and zebrafish is not embryonically lethal owing to maternal *Smn* contribution (Box 1) (Boon et al., 2009; Briese et al., 2008; Chan et al., 2003), providing the opportunity to develop models in which no functional Smn is present at later developmental stages. Moreover, knockdown of *Smn* using genetic tools, such as morpholinos, can be used to transiently reduce Smn levels in zebrafish (Boon et al., 2009; McWhorter et al., 2003). Finally, in animal models in which modification of the genome is challenging, such as pigs, transient knockdown of *Smn1* has been induced by adeno-associated virus (AAV; Box 1) 9-mediated delivery of specific short harpin RNAs (shRNAs) (Duque et al., 2015). In invertebrate models of SMA, synaptic defects, studied in *C. elegans* (Briese et al., 2008; Doyle et al., 2020), and altered NMJ development, studied in *Drosophila* (Chan et al., 2003), lead to defective locomotion and reduced lifespan as the most prominent disease phenotype. Moreover, *Drosophila* are relatively suitable for studying intermediate SMA phenotypes, as *Smn* loss-of-function mutations can be used to reliably generate animals with varying degrees of phenotypic severity and survival (Spring et al., 2019). Similarly, in zebrafish, knockdown of Smn1 leads to varying degrees of defects in axon and NMJ development (Boon et al., 2009; McWhorter et al., 2003) and locomotion behaviour (Tay et al., 2021). As zebrafish are optically transparent during development, the use of this model contributed significantly to our understanding of the effect of low levels of Smn1 on nervous system development (Boon et al., 2009; McWhorter et al., 2003). In addition to these phenotypes, pigs provided an opportunity to study neurophysiological characteristics upon SMN reduction, showing similar changes to those observed in patients (Duque et al., 2015).

Mouse models have been especially critical in the development of current therapies and to study the roles of the SMN protein (Table 2) (Lauria et al., 2020; Sleigh et al., 2011b). Genetically, most mouse models for SMA are based on genetic knockout of the *Smn1* gene and the introduction of a second *SMN2*-like allele. Commonly used models include those based on *SMN2* transgenes (Burghes and Taiwanese models; Monani et al., 2000; Hsieh-Li et al., 2000), *SMN2* with the addition of an *SMN*Δ*7* allele (Δ7 model; Le et al., 2005), and models based on the mouse *Smn2B* allele that drives alternative splicing of *Smn1* exon 7 (Bowerman et al., 2012a). Phenotypically, most mouse models of SMA are characterised by lower body weight, reduced survival and changes in locomotor behaviour, such as changes in the righting reflex (Box 1) in neonatal mice (Butchbach et al., 2007). Pathologically, they are characterised by varying degrees of motor neuron loss, NMJ pathology and muscle defects (Le et al., 2005; Monani et al., 2000; Murray et al., 2008). In addition to varying motor pathologies, systemic pathologies affecting almost all organs have also been described in mice (Allardyce et al., 2020; Deguise et al., 2019; Šoltić et al., 2019; Thomson et al., 2017). The severity of such systemic, non-CNS pathology, however, varies extensively between mouse models and depends, at least partially, on the genetic background of the mouse model (Buettner et al., 2021). The extensive genetic toolbox available to modify the mouse genome, including models based on the bacterial recombinase Cre and models with inducible alleles, has been particularly useful in improving our understanding of the developmental and tissue requirements for SMN expression. For example, studies using these tools have shown that SMN expression is required throughout the neuromuscular system (Fletcher et al., 2017; Hunter et al., 2016; Kim et al., 2020; Park et al., 2010) and that sufficient levels of SMN expression are critical during a narrow time window in postnatal development (Kariya et al., 2014; Lutz et al., 2011).

The central contribution of SMA models to the development of SMA therapies was instigated by the fact that they reflected key features of severe SMA: a short lifespan and severe motor neuron pathology that could be (partially) rescued by restoring SMN expression. However, the current shift in SMA phenotypes caused by the treatment of many patients at various symptomatic and presymptomatic stages calls for disease models that accurately reflect this development.

## Limitations of existing models

To allow us to assess the characteristics that novel SMA disease models should ideally have, we will first discuss some specific limitations of current models. Although some of these points are general limitations of using other species to model human disease, we highlight them because of relevance in the light of recent developments in clinical SMA research.

### Limitations of current small animal models

The functional domains of SMN are highly conserved across species (the amino acid homology ranges from 25% identity in *C. elegans* to 53% in zebrafish) and *Smn* depletion leads to locomotor defects and neuromuscular phenotypes in most species. However, when studying the conservation of SMN function across species, the expression of zebrafish *smn1* but not *Drosophila Smn* or *C. elegans smn-1* was able to improve the phenotype of *SMN*Δ*7* mice (Osman et al., 2019). This indicates that despite similarities, evolutionarily and functionally, not all SMN genes are equal. When using small animal models, it is also important to consider the widely different anatomy and structure of their neuromuscular systems compared to those of humans. In particular, the metamorphosis process that *Drosophila* undergoes is not in any way comparable to the development of neuromuscular systems in vertebrates. Finally, using common genetic tools, it is challenging to generate these models, as discussed in the previous section, to draw comparisons to the inverted duplication that makes up the *SMN* locus in the human genome, with limitations comparable to those in mouse models, as discussed in the next section.

### Genetics of SMA mouse models

Because only humans possess *SMN2*, several innovative genetic approaches have been used to generate SMA animal models that survive past embryogenesis. However, the transcription and translation of *SMN* in models that rely on human transgenes (e.g. *SMN2* and *SMN2-SMN2*Δ*7*) may not be physiologically regulated, owing to differences between mouse and human genetic elements that regulate expression, and to variation in insertion sites of transgenes between models. Similarly, regulatory elements, such as the long non-coding RNA *SMN-AS1* (Box 1) (d'Ydewalle et al., 2017), are thought to be human-specific, and studying their function in mouse models may therefore be challenging. Although subtle, these differences may complicate comprehensively answering important questions, such as how much SMN protein is required by different tissues at varying developmental time points.

As has already been observed in mouse models of other neuromuscular diseases, including amyotrophic lateral sclerosis (Heiman-Patterson et al., 2005) and Duchenne muscular dystrophy (Coley et al., 2016), the genetic background of mouse strains can be an important determinant of the observed phenotype. In SMA, the Taiwanese and 2B mouse models have a less severe phenotype, with longer survival and reduced peripheral pathology, when the mice are on a C57BL/6 background compared to an FVB background (Buettner et al., 2021; Hosseinibarkooie et al., 2016) (Table 2). A possible explanation for this difference is the altered expression of modifier genes (Box 1); for instance, in the spinal cord of 2B mice on a C57BL/6 background, the expression of plastin-3 (*Pls3*) – a positive modifier gene in patients of specific families – was significantly increased compared to the same model on an FVB background (Eshraghi et al., 2016). This highlights the importance of careful reporting of the genetic background for correct interpretation of the results from studies using such inbred strains.

### SMN protein requirements in patients and models

Although *SMN2* is often suggested to produce ∼10% of functional SMN protein when compared to *SMN1*, animal and post-mortem studies suggest that this is substantially more nuanced and dependent on many factors, including age (or developmental stage) and the tissue or cell type analysed (Groen et al., 2018b; Wadman et al., 2016). The only study to robustly determine SMN protein and RNA levels in a large number of post-mortem spinal cord samples, from unaffected individuals and patients with and without SMA, found that SMN levels were especially high in prenatal controls, followed by a quick decline after birth (Ramos et al., 2019). They also found that SMN levels were substantially lower in patients with SMA both prenatally and neonatally, but that later in life, the expression levels were often comparable to those in unaffected individuals. However, the stability of SMN protein levels throughout life is challenging to study as repeated sampling of disease-relevant tissues is impossible. Studies in more accessible tissues suggest that SMN levels may decline further throughout life in patients with SMA (Ramos et al., 2019; Wadman et al., 2016) or remain relatively stable – especially in adults – depending on the cell type studied (Groen et al., 2018b; Kobayashi et al., 2011; Zaworski et al., 2016).

The most severe mouse models of SMA are characterised by a very progressive phenotype and low levels of the SMN protein. The development of models that mimic milder forms of SMA, and are thus based on higher levels of SMN expression, is therefore especially important. An allelic series of mouse models provided some interesting insights into the challenges associated with developing such models (Bogdanik et al., 2015; Osborne et al., 2012). In these studies, varying combinations of mouse *Smn1* mutants and human *SMN2* transgenes led to phenotypes that were either extremely severe (embryonically lethal) or relatively mild (long survival) and showed that the absence or presence of one extra, partially functional copy of an SMN gene was sufficient for this sudden switch in phenotype severity. This is different from the broad range of phenotypes in patients in which normal development can occur with symptom onset as late as >30 years of age, for example, in higher *SMN2* copy number carriers (Piepers et al., 2008). In general, the development of disease models that accurately reflect relatively milder forms of SMA has not been straightforward, suggesting that in mice, the range of variation in SMN protein expression may be narrower than in humans (O'Hern et al., 2017), complicating the generation of models with intermediate phenotypes required for studying milder forms of SMA.

### Treatment delivery in animal models

Before the start of clinical trials, the efficacy of SMN-targeting treatments was well established in animal models (Hua et al., 2011; Poirier et al., 2018; Valori et al., 2010). A major difference between currently approved treatments and those developed at preclinical stages are the delivery routes used for these medicines, which are difficult to replicate in cellular and animal models. For example, in humans, the blood-brain barrier is more permeable after birth than later in life and this permeability possibly persists up to 6 months after birth (Lam et al., 2015). In mice, this is the case for several days (possibly up to postnatal day 10) (Coelho–Santos and Shih, 2020), which overlaps with the critical time window for NMJ development, thus complicating a reliable comparison of insights from animal models and patients. In general, it is difficult to compare the biodistribution of different AAV serotypes between species and care should be taken in extrapolating observations between species. Studies in non-human primates were therefore instrumental in improving our understanding of the possibilities and limitations of using different AAV serotypes for therapy delivery (Davidoff et al., 2005; Foust et al., 2009) and may remain critical for the development of future AAV-based treatment, including base-editing strategies (Arbab et al., 2023). In a single post-mortem study of two cases, onasemnogene abeparvovec distribution was observed systemically, although viral copy number per cell varied extensively (up to 1000-fold) between tissues and cell types (Thomsen et al., 2021). For ASO treatment, although intracerebroventricular injection in mice mimics the delivery of ASOs into the cerebrospinal fluid of patients (Chiriboga et al., 2016), the final distribution of the treatment may still differ. Indeed, in limited post-mortem analyses of patients receiving nusinersen treatment, ASO distribution appeared to be restricted to mostly the lower segments of the spinal cord, near the lumbar injection site (Ramos et al., 2019). It should be noted, however, that in post-mortem cases, treatment effects may have been especially limited and therefore do not completely reflect common treatment effects. If and how it is feasible to better recapitulate treatment delivery in disease models remains questionable, but should be considered when translating model findings into the clinic.

### Non-CNS phenotypes

Especially in mouse models of SMA, many non-CNS phenotypes have been described (see Table 2), including heart, gastrointestinal, liver, pancreas, kidney and vascular defects (Hamilton and Gillingwater, 2013; Yeo and Darras, 2020). Many of these phenotypes have also been reported in patients, although often analyses were restricted to post-mortem tissues of untreated patients (Hamilton and Gillingwater, 2013; Yeo and Darras, 2020), as the ability to study organ function in patients is substantially more limited than in animal models. To what extent the non-CNS phenotypes observed in disease models are relevant to patient symptoms remains to be further investigated, although differences are likely. For example, in mice with mild and severe SMA phenotypes, skin necrosis of tails and ears is commonly observed, whereas in patients, necrosis has been reported only in a limited number of very severe cases with one *SMN2* copy (Weissman et al., 2021). Moreover, SMA mice often do not die from progressive neuromuscular decline but rather die abruptly, usually from heart defects (Bogdanik et al., 2015). In contrast, in patients, major heart impairments have only been reported in limited numbers of patients that are generally characterised by a severe phenotype and neonatal onset (Palladino et al., 2011; Rudnik-Schöneborn et al., 2008; Wijngaarde et al., 2017). These and other discrepancies can be due to varying reasons, including different roles or requirements of SMN, or alternative expression patterns of SMN in transgenic mice that remain to be identified. However, it is important to remember that these non-CNS phenotypes may appear during long-term treatment follow-up of patients with SMA, especially in patients that receive CNS-specific treatment of SMA. The extent and type of non-CNS pathology may again lead to a more variable phenotype in patients, depending on initial disease severity and type and start of treatment.

In summary, many factors including genetics, protein expression regulation, treatment delivery routes and non-CNS phenotypes may limit the use of current animal models to study important aspects of SMA in the therapeutic era. However, discussing these points may lead to insights that can facilitate the development and improvement of novel and existing model systems going forward.

## Future development of models for the therapeutic era

### Future development of small animal models for SMA

*Drosophila* and *C. elegans* have been particularly useful for large-scale screening of drugs or genetic knockdown libraries *in vivo* (Aquilina and Cauchi, 2018; De Carlos Cáceres et al., 2018) and to study the effect of specific modifier genes (Hensel et al., 2021), and are a useful part of the SMA research toolbox that can be harnessed further. The transparency of *C. elegans*, combined with a detailed knowledge of number, location and connections of all (motor) neurons, allows for detailed analyses that are not always possible in other species.

In more complex small vertebrates, such as zebrafish, optimisation of screening and imaging tools increasingly enables high-throughput screening. For example, zebrafish were used to screen the effect of a library of synapse stabilisation compounds for SMA using a semi-automated imaging platform (Oprişoreanu et al., 2021). A similar platform might be used in the future for screening across a broad range of non-neuronal phenotypes by transgenic labelling of specific cell types. Moreover, owing to their high-throughput capabilities and genetic tractability, zebrafish have been and can be used to identify and investigate (genetic) modifiers and their role in influencing disease severity (Akten et al., 2011; Boyd et al., 2017; Lotti et al., 2012; Oprea et al., 2008; Powis et al., 2016; Riessland et al., 2017; Wishart et al., 2014). The use of tools, such as precise CRISPR/Cas9-mediated genome editing, in zebrafish will further extend and enhance the possibilities to perform these kinds of analyses (Ding et al., 2023).

### Further development and refinement of mouse models for SMA

Despite numerous challenges in using mouse models to mimic important features of SMA, the genetic toolbox available for mouse model development is extensive and has not yet been exhausted for SMA research. A perhaps obvious but important addition to the current palette of SMA models would be the generation of models based on outbred lines, such as the JAX diversity outbred mouse population (Lutz, 2018). The current use of inbred strains prohibits the possibility to model the genetic variation that occurs in real-world patient populations and is therefore more like studying a small group of comparable patients. Even though the use of outbred models may initially require an increased number of animals and concurrent ethical discussions, observations about SMA pathogenesis and treatment effects may ultimately prove to be more translatable when population genetics have been considered at the modelling stage.

Important insights into the spatial and temporal requirements of SMN, especially in the neuromuscular system, have been obtained by crossing existing SMA models with cell-type-specific or inducible-Cre mouse lines (Kariya et al., 2014; Kim et al., 2020; Lutz et al., 2011; Sleigh et al., 2011b). This approach will still be useful in the future, as the role of SMN in different organs and during development remains incompletely understood, and specific Cre lines for many cell types are readily available to investigate this further. This would also allow the study of the effect of rescuing SMN expression specifically in the CNS, as is currently the case with nusinersen treatment, compared to systemic approaches. Cre-based models also provide powerful tools to investigate the role of the SMN protein in specific tissues and cell types, at various time points, and independent of SMA pathology.

An important challenge for further mouse model development is the refinement of SMN protein expression to enable reliable modelling of milder forms of SMA. Some approaches for this already exist but have had comparatively little investigation, such as models based on milder mutations in *SMN1* and treatment-based approaches (Monani et al., 2003; Sahashi et al., 2013; Workman et al., 2009; Zhou et al., 2015). The latter would, for example, include approaches that use suboptimal dosing of an SMN-targeting ASO to partially restore SMN levels and improve mouse lifespan. Although this is a valuable approach, in practice, it is not straightforward to achieve consistent expression across mice and between litters. The former would include the generation of mouse lines based on mutations in *SMN1* that are known to be associated with relatively milder forms of SMA, such as p.S230L (Nölle et al., 2011) and p.G279C (Sharifi et al., 2021). The advantage of this approach would be that it may be possible to generate mouse models without the addition of a human transgene, which is comparable to the current 2B/− model (Bowerman et al., 2012a), so that SMN expression is more physiologically regulated.

By considering the remaining possibilities for mouse models, they are likely to keep their central role in SMA research for years to come.

### Large animal models for SMA

The NMJ of sheep and pigs resembles the human NMJ much more closely than that of rats or mice (Boehm et al., 2020). However, the development of SMA models based on these larger animals still remains relatively new. A pig model of SMA, based on SMN knockdown using a virus expressing an shRNA targeting pig *SMN1*, has previously been published (Duque et al., 2015). This model allowed the characterisation of the postnatal role of SMN in motor neurons and the evaluation of clinically relevant biomarkers, such as compound muscle action potential (CMAP) and motor unit number estimation (MUNE), in response to SMN restoration. This represents an advantage of working with large animal models, as neurophysiological biomarkers may be more difficult to investigate in smaller animal models. Additionally, pigs possess several characteristics that are considerably more comparable to those of humans, such as their cardiovascular system, digestive tract and cytochrome P450 enzymes, (Ribitsch et al., 2020; Schelstraete et al., 2019), which imply that the outcomes of drug metabolism and toxicity studies from pigs would be more translatable to humans. In addition to obvious logistical challenges around working with large animal models, genetic manipulation is generally not straightforward. In other diseases, spontaneous mutations in disease-relevant genes have been used successfully to study diseases such as Batten's disease in sheep (Frugier et al., 2008). However, due to the absence of *SMN2* in other species, spontaneous *SMN1* mutations would most likely also be embryonically lethal. Should it become possible to genetically edit pigs or sheep to mimic SMA, it would provide an important, disease-relevant readout for further analysis and translatability to the clinic.

### Patient-derived cellular models

A remaining challenge when using animal models for SMA is the fact that *SMN2* is specific to humans, which will always limit the specificity of animal models in SMA research. Advances in *in vitro* disease model systems in recent years have, however, been significant and provide important steps forward. An additional advantage of using patient-derived cellular models of SMA is the ability to include a diverse genetic population in analyses, thereby bypassing an important limitation of having to use many inbred mouse models as discussed above. Moreover, human models would allow us to better study the physiological regulation of *SMN2* expression in more detail. We will discuss several options that may be of particular use, depending on the type of experimental design required and the goal of specific analyses.

One of the most basic patient-derived cellular models are primary fibroblasts. Fibroblasts are readily obtained from skin biopsies from patients and can be collected, cultured and analysed from large numbers of patients. In addition, many researchers in the SMA field have obtained fibroblast lines from public biobanks, such as the Coriell repository (Adami and Bottai, 2019). Although the morphological and functional difference between fibroblasts and motor neurons is substantial, fibroblasts do reflect key features of SMA, such as reduced *SMN2* RNA and SMN protein levels, and respond well to *in vitro* treatments (Garbes et al., 2013; Singh et al., 2006; Wadman et al., 2016). Moreover, primary patient-derived fibroblasts can be used without further modifications, therefore preserving (epi)genetic variants that may be putative modifiers of SMA outcomes. For example, within families, *SMN2* copy number is often equal, whereas SMA severity often is not – one possible explanation for this discrepancy may be alternative methylation of the *SMN2* copies between siblings (Hauke et al., 2009; Wadman et al., 2020). However, when aiming to preserve epigenetic variants, fast expansion of low-passage cultures into the number of cells required for the experiment is critical, as, in high-passage cultures, DNA methylation changes over time (Franzen et al., 2021; Koch et al., 2011).

Since the Yamanaka paper in 2006 (Takahashi and Yamanaka, 2006), many steps have been taken to refine the differentiation of patient-derived primary cells, such as fibroblasts, into a pluripotent stem cell state. The generation of such induced pluripotent stem cells (iPSCs) provides an exceptionally powerful tool, as it allows scientists to subsequently differentiate these into practically any cell type of their choice. Indeed, many groups in the SMA field have used this approach to study the effect of low SMN levels on iPSC-derived motor neuron function. Currently published studies, however, have some limitations. Many groups are using one or several of a limited number of Coriell repository cell lines as a starting point for their analyses (Adami and Bottai, 2019), largely bypassing the opportunity to capture as much genetic variability as possible and with the risk of missing relevant variation in phenotype or treatment effect (Garbes et al., 2013). Extensive characterisation of primary patient-derived fibroblasts from various biobanks will support optimal selection of representative patient cell lines, based on SMA type, genetics and therapy response, when generating iPSC lines for further study. Moreover, the SMN protein has been suggested to play an important role in the differentiation process from primary fibroblast to an iPSC state (Chang et al., 2022; Varderidou-Minasian et al., 2021), which may cause additional variation after differentiation. Alternatively, for SMA research, direct conversion approaches (see e.g. Ladewig et al., 2012) may be particularly promising, as this allows fibroblasts to be differentiated into neurons without the need for a pluripotent intermediate state. Here, the main limitation will be the lack of a cell expansion step comparable to iPSC culture, limiting the amount of material that can be generated in single experiments. Still, the use of iPSC-derived motor neurons, for example, in co-culture with iPSC-derived myotubes to generate *in vitro* NMJ systems (Faustino Martins et al., 2020; de Jongh et al., 2021; Southam et al., 2013), holds great future promise for detailed analyses of treatment effects and molecular analyses at various important cellular sites. Comparable to how Cre-based mouse models have been used to characterise the requirements for SMN expression in different parts of the neuromuscular system, these *in vitro* NMJ systems allow the study of these requirements in different patient-derived cell types. Moreover, they can be used to gain valuable insights on the local mechanistic effects of reduced SMN expression. Lastly, they can be used to study the effect of treatment when targeted to the entire neuromuscular system or, in contrast, when restricted to specific parts only.

Finally, many research groups around the globe are developing increasingly complex organoid systems to model a range of complex diseases. The use of organoids in the SMA field has so far been relatively limited, as spinal organoids do not as reliably reflect spinal cord anatomy and development as, for example, brain organoids mimic cortical development and anatomy (Hor et al., 2018). However, current advances in culturing multi-modal organoids, for example, systems that connect cortical, spinal and muscle organoids, essentially mimicking the entire neuromuscular system in a dish (Andersen et al., 2020), illustrate the increasing versatility of complex culture systems. This opens up many new opportunities for research into local treatment effects and tissue-specific pathological changes that may occur in SMA, in line with *in vitro* NMJ systems as discussed above. Additionally, multi-organoid systems that have been developed to study, for example, the interaction between the kidney and liver (Nguyen et al., 2022) may provide a great possibility to study non-CNS phenotypes of SMA in detail, including targeting treatments to such phenotypes. So far, most of our knowledge on non-CNS phenotypes in patients with SMA is based on limited post-mortem studies. Therefore, the opportunity to use patient-derived organoid cultures to gain a more in-depth understanding of the consequences of low levels of SMN on organ function is highly promising. Although challenging to set up, concurrently culturing different organoids in separate compartments of the same culture system provides a fascinating opportunity to study the interaction and signalling between organoids. This also enables the comparison of treatment effects when it is either targeted to one organoid or the whole system.

In summary, even though the SMA field has already benefitted from the availability of many useful disease models, several routes for further optimisation and development are still available. Efficiently combining multiple model systems – including small animal and cellular models for high-throughput screening, mouse models to study molecular mechanisms, large animal models for biomarker discovery and drug development, and patient-derived models to better study patient heterogeneity and SMN regulation – holds great promise, both in terms of scalability and inclusion of genetic variation, as well as the detailed study of local pathological changes and treatment effects.

## Concluding remarks

**Fig. 2.**
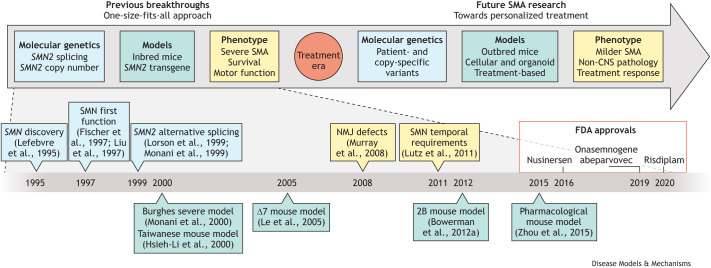
**Summary of current and future SMA model focus and characteristics.** Initial developments after the discovery of the genetic cause of SMA included further breakthroughs in the molecular genetic characterisation of SMA (Fischer et al., 1997; Liu et al., 1997) and the generation of key mouse models, through which central SMA phenotypes were interrogated. This led to the current gene-targeting strategies to increase SMN levels in patients. A selection of important milestones are indicated at the bottom of the figure along with the years of initial US Food and Drug Administration (FDA) approval of current gene-targeting therapies for nusinersen, onasemnogene abeparvovec and risdiplam. To investigate the limitations of current treatments and further our understanding of heterogeneity in SMA and treatment outcomes, new disease models are needed that better take into account the important characteristics of SMA and provide a starting point for future personalisation of SMA treatment. CNS, central nervous system; NMJ, neuromuscular junction; SMA, spinal muscular atrophy; SMN, survival of motor neuron.

The SMA research field is changing rapidly. The approval of multiple gene-targeting treatments and their optimal use through early, often presymptomatic treatment provides a significant step forward for patients with SMA and their families. The observed heterogeneity in both disease severity and treatment outcomes, however, highlights that the current ‘one-size-fits-all’ *SMN*-targeting treatment approach may, in the future, need to be replaced by more individualised approaches (Fig. 2). Without the continuous further development and investigation of current and new SMA models, it will not be possible to develop such individualised treatment approaches. Although this Review has been speculative at times, we have highlighted several features to consider when contemplating the use and further development of various SMA models in the future. We hope that this will help the field to determine the important next steps that need to be taken to advance our understanding of SMA further and keep improving the lives of all those affected by SMA.

## References

[DMM050352C1] Adami, R. and Bottai, D. (2019). Spinal muscular atrophy modeling and treatment advances by induced pluripotent stem cells studies. *Stem. Cell Rev. Rep.* 15, 795-813. 10.1007/s12015-019-09910-6 31863335

[DMM050352C2] Akten, B., Kye, M. J., Hao, L. T., Wertz, M. H., Singh, S., Nie, D., Huang, J., Merianda, T. T., Twiss, J. L., Beattie, C. E. et al. (2011). Interaction of survival of motor neuron (SMN) and HuD proteins with mRNA cpg15 rescues motor neuron axonal deficits. *Proc. Natl. Acad. Sci. USA* 108, 10337-10342. 10.1073/pnas.1104928108 21652774 PMC3121858

[DMM050352C3] Allardyce, H., Kuhn, D., Hernandez-Gerez, E., Hensel, N., Huang, Y.-T., Faller, K., Gillingwater, T. H., Quondamatteo, F., Claus, P. and Parson, S. H. (2020). Renal pathology in a mouse model of severe Spinal Muscular Atrophy is associated with downregulation of Glial Cell-Line Derived Neurotrophic Factor (GDNF). *Hum. Mol. Genet.* 29, 2365-2378. 10.1093/hmg/ddaa126 32588893

[DMM050352C4] Andersen, J., Revah, O., Miura, Y., Thom, N., Amin, N. D., Kelley, K. W., Singh, M., Chen, X., Thete, M. V., Walczak, E. M. et al. (2020). Generation of functional human 3D cortico-motor assembloids. *Cell* 183, 1913-1929.e26. 10.1016/j.cell.2020.11.017 33333020 PMC8711252

[DMM050352C5] Antonaci, L., Pera, M. C. and Mercuri, E. (2023). New therapies for spinal muscular atrophy: where we stand and what is next. *Eur. J. Pediatr* 182, 2935-2942. 10.1007/s00431-023-04883-8 37067602 PMC10354145

[DMM050352C6] Aquilina, B. and Cauchi, R. J. (2018). Genetic screen identifies a requirement for SMN in mRNA localisation within the Drosophila oocyte. *BMC Res. Notes* 11, 378. 10.1186/s13104-018-3496-1 29895323 PMC5998591

[DMM050352C7] Arbab, M., Matuszek, Z., Kray, K. M., Du, A., Newby, G. A., Blatnik, A. J., Raguram, A., Richter, M. F., Zhao, K. T., Levy, J. M. et al. (2023). Base editing rescue of spinal muscular atrophy in cells and in mice. *Science* 380, eadg6518. 10.1126/science.adg6518 36996170 PMC10270003

[DMM050352C8] Arnold, W. D., Porensky, P. N., McGovern, V. L., Iyer, C. C., Duque, S., Li, X., Meyer, K., Schmelzer, L., Kaspar, B. K., Kolb, S. J. et al. (2014). Electrophysiological biomarkers in spinal muscular atrophy: proof of concept. *Ann. Clin. Transl. Neurol.* 1, 34-44. 10.1002/acn3.23 24511555 PMC3914317

[DMM050352C9] Baranello, G., Darras, B. T., Day, J. W., Deconinck, N., Klein, A., Masson, R., Mercuri, E., Rose, K., El-Khairi, M., Gerber, M. et al. (2021). Risdiplam in type 1 spinal muscular atrophy. *N. Engl. J. Med.* 384, 915-923. 10.1056/NEJMoa2009965 33626251

[DMM050352C10] Bevan, A. K., Hutchinson, K. R., Foust, K. D., Braun, L., McGovern, V. L., Schmelzer, L., Ward, J. G., Petruska, J. C., Lucchesi, P. A., Burghes, A. H. M. et al. (2010). Early heart failure in the SMNΔ7 model of spinal muscular atrophy and correction by postnatal scAAV9-SMN delivery. *Hum. Mol. Genet.* 19, 3895-3905. 10.1093/hmg/ddq300 20639395 PMC2947399

[DMM050352C11] Boehm, I., Alhindi, A., Leite, A. S., Logie, C., Gibbs, A., Murray, O., Farrukh, R., Pirie, R., Proudfoot, C., Clutton, R. et al. (2020). Comparative anatomy of the mammalian neuromuscular junction. *J. Anat.* 237, 827-836. 10.1111/joa.13260 32573802 PMC7542190

[DMM050352C12] Bogdanik, L. P., Osborne, M. A., Davis, C., Martin, W. P., Austin, A., Rigo, F., Bennett, C. F. and Lutz, C. M. (2015). Systemic, postsymptomatic antisense oligonucleotide rescues motor unit maturation delay in a new mouse model for type II/III spinal muscular atrophy. *Proc. Natl. Acad. Sci. USA* 112, E5863-E5872. 10.1073/pnas.1509758112 26460027 PMC4629342

[DMM050352C13] Boon, K.-L., Xiao, S., McWhorter, M. L., Donn, T., Wolf-Saxon, E., Bohnsack, M. T., Moens, C. B. and Beattie, C. E. (2009). Zebrafish survival motor neuron mutants exhibit presynaptic neuromuscular junction defects. *Hum. Mol. Genet.* 18, 3615-3625. 10.1093/hmg/ddp310 19592581 PMC2742401

[DMM050352C14] Bowerman, M., Anderson, C. L., Beauvais, A., Boyl, P. P., Witke, W. and Kothary, R. (2009). SMN, profilin IIa and plastin 3: A link between the deregulation of actin dynamics and SMA pathogenesis. *Mol. Cell. Neurosci.* 42, 66-74. 10.1016/j.mcn.2009.05.009 19497369

[DMM050352C15] Bowerman, M., Murray, L. M., Beauvais, A., Pinheiro, B. and Kothary, R. (2012a). A critical smn threshold in mice dictates onset of an intermediate spinal muscular atrophy phenotype associated with a distinct neuromuscular junction pathology. *Neuromuscul. Disord.* 22, 263-276. 10.1016/j.nmd.2011.09.007 22071333

[DMM050352C16] Bowerman, M., Swoboda, K. J., Michalski, J.-P., Wang, G.-S., Reeks, C., Beauvais, A., Murphy, K., Woulfe, J., Screaton, R. A., Scott, F. W. et al. (2012b). Glucose metabolism and pancreatic defects in spinal muscular atrophy. *Ann. Neurol.* 72, 256-268. 10.1002/ana.23582 22926856 PMC4334584

[DMM050352C17] Boyd, P. J., Tu, W.-Y., Shorrock, H. K., Groen, E. J. N., Carter, R. N., Powis, R. A., Thomson, S. R., Thomson, D., Graham, L. C., Motyl, A. A. L. et al. (2017). Bioenergetic status modulates motor neuron vulnerability and pathogenesis in a zebrafish model of spinal muscular atrophy. *PLoS Genet.* 13, e1006744. 10.1371/journal.pgen.1006744 28426667 PMC5417717

[DMM050352C18] Briese, M., Esmaeili, B., Fraboulet, S., Burt, E. C., Christodoulou, S., Towers, P. R., Davies, K. E. and Sattelle, D. B. (2008). Deletion of smn-1, the Caenorhabditis elegans ortholog of the spinal muscular atrophy gene, results in locomotor dysfunction and reduced lifespan. *Hum. Mol. Genet.* 18, 97-104. 10.1093/hmg/ddn320 18829666 PMC2644645

[DMM050352C19] Buettner, J. M., Sime Longang, J. K., Gerstner, F., Apel, K. S., Blanco-Redondo, B., Sowoidnich, L., Janzen, E., Langenhan, T., Wirth, B. and Simon, C. M. (2021). Central synaptopathy is the most conserved feature of motor circuit pathology across spinal muscular atrophy mouse models. *iScience* 24, 103376. 10.1016/j.isci.2021.103376 34825141 PMC8605199

[DMM050352C20] Butchbach, M. E. R., Edwards, J. D. and Burghes, A. H. M. (2007). Abnormal motor phenotype in the SMNΔ7 mouse model of spinal muscular atrophy. *Neurobiol. Dis.* 27, 207-219. 10.1016/j.nbd.2007.04.009 17561409 PMC2700002

[DMM050352C21] Butchbach, M. E. R., Rose, F. F., Rhoades, S., Marston, J., McCrone, J. T., Sinnott, R. and Lorson, C. L. (2010). Effect of diet on the survival and phenotype of a mouse model for spinal muscular atrophy. *Biochem. Biophys. Res. Commun.* 391, 835-840. 10.1016/j.bbrc.2009.11.148 19945425 PMC2839161

[DMM050352C22] Calucho, M., Bernal, S., Alías, L., March, F., Venceslá, A., Rodríguez-Álvarez, F. J., Aller, E., Fernández, R. M., Borrego, S., Millán, J. M. et al. (2018). Correlation between SMA type and SMN2 copy number revisited: An analysis of 625 unrelated Spanish patients and a compilation of 2834 reported cases. *Neuromuscul. Disord.* 28, 208-215. 10.1016/j.nmd.2018.01.003 29433793

[DMM050352C151] Campagne, S., Boigner, S., Rüdisser, S., Moursy, A., Gillioz, L., Knörlein, A., Hall, J., Ratni, H., Cléry, A. and Allain, F. H. (2019). Structural basis of a small molecule targeting RNA for a specific splicing correction. *Nat. Chem. Biol.* 15, 1191-1198. 10.1038/s41589-019-0384-5 31636429 PMC7617061

[DMM050352C23] Chan, Y. B., Miguel-Aliaga, I., Thomas, N., Trülzsch, B., Sattelle, D. B., Davies, K. E. and van der Heuvel, M. (2003). Neuromuscular defects in a Drosophila survival motor neuron gene mutant. *Hum. Mol. Genet.* 12, 1367-1376. 10.1093/hmg/ddg157 12783845

[DMM050352C24] Chang, H. C.-H., Dimlich, D. N., Yokokura, T., Mukherjee, A., Kankel, M. W., Sen, A., Sridhar, V., Fulga, T. A., Hart, A. C., Van Vactor, D. et al. (2008). Modeling spinal muscular atrophy in drosophila. *PLoS ONE* 3, e3209. 10.1371/journal.pone.0003209 18791638 PMC2527655

[DMM050352C25] Chang, W.-F., Lin, T.-Y., Peng, M., Chang, C.-C., Xu, J., Hsieh-Li, H.-M., Liu, J.-L. and Sung, L.-Y. (2022). Survival motor neuron enhances pluripotent gene expression and facilitates cell reprogramming. *Stem Cells Dev.* 31, 696-705. 10.1089/scd.2022.0091 35848514

[DMM050352C26] Chaytow, H., Faller, K. M. E., Huang, Y.-T. and Gillingwater, T. H. (2021). Spinal muscular atrophy: From approved therapies to future therapeutic targets for personalized medicine. *Cell Rep. Med.* 2, 100346. 10.1016/j.xcrm.2021.100346 34337562 PMC8324491

[DMM050352C27] Chiriboga, C. A., Swoboda, K. J., Darras, B. T., Iannaccone, S. T., Montes, J., De Vivo, D. C., Norris, D. A., Bennett, C. F. and Bishop, K. M. (2016). Results from a phase 1 study of nusinersen (ISIS-SMN_Rx_) in children with spinal muscular atrophy. *Neurology* 86, 890-897. 10.1212/WNL.0000000000002445 26865511 PMC4782111

[DMM050352C28] Cobb, M. S., Rose, F. F., Rindt, H., Glascock, J. J., Shababi, M., Miller, M. R., Osman, E. Y., Yen, P.-F., Garcia, M. L., Martin, B. R. et al. (2013). Development and characterization of an SMN2-based intermediate mouse model of Spinal Muscular Atrophy. *Hum. Mol. Genet.* 22, 1843-1855. 10.1093/hmg/ddt037 23390132

[DMM050352C29] Coelho–Santos, V. and Shih, A. Y. (2020). Postnatal development of cerebrovascular structure and the neurogliovascular unit. *WIREs Dev. Biol.* 9, e363. 10.1002/wdev.363 PMC7027551 31576670

[DMM050352C30] Coley, W. D., Bogdanik, L., Vila, M. C., Yu, Q., Van Der Meulen, J. H., Rayavarapu, S., Novak, J. S., Nearing, M., Quinn, J. L., Saunders, A. et al. (2016). Effect of genetic background on the dystrophic phenotype in *mdx* mice. *Hum. Mol. Genet.* 25, 130-145. 10.1093/hmg/ddv460 26566673 PMC4690497

[DMM050352C152] Crooke, S. T., Wang, S., Vickers, T. A., Shen, W. and Liang, X. H. (2017). Cellular uptake and trafficking of antisense oligonucleotides. *Nat. Biotechnol.* 35, 230-237. 10.1038/nbt.3779 28244996

[DMM050352C31] d'Ydewalle, C., Ramos, D. M., Pyles, N. J., Ng, S.-Y., Gorz, M., Pilato, C. M., Ling, K., Kong, L., Ward, A. J., Rubin, L. L. et al. (2017). The antisense transcript SMN-AS1 regulates SMN expression and is a novel therapeutic target for spinal muscular atrophy. *Neuron* 93, 66-79. 10.1016/j.neuron.2016.11.033 28017471 PMC5223741

[DMM050352C32] Dangouloff, T., Vrščaj, E., Servais, L., Osredkar, D., Adoukonou, T., Aryani, O., Barisic, N., Bashiri, F., Bastaki, L., Benitto, A. et al. (2021). Newborn screening programs for spinal muscular atrophy worldwide: Where we stand and where to go. *Neuromuscul. Disord.* 31, 574-582. 10.1016/j.nmd.2021.03.007 33985857

[DMM050352C33] Davidoff, A. M., Gray, J. T., Ng, C. Y. C., Zhang, Y., Zhou, J., Spence, Y., Bakar, Y. and Nathwani, A. C. (2005). Comparison of the ability of adeno-associated viral vectors pseudotyped with serotype 2, 5, and 8 capsid proteins to mediate efficient transduction of the liver in murine and nonhuman primate models. *Mol. Ther.* 11, 875-888. 10.1016/j.ymthe.2004.12.022 15922958

[DMM050352C34] De Carlos Cáceres, I., Porto, D. A., Gallotta, I., Santonicola, P., Rodríguez-Cordero, J., Di Schiavi, E. and Lu, H. (2018). Automated screening of *C. elegans* neurodegeneration mutants enabled by microfluidics and image analysis algorithms. *Integr. Biol.* 10, 539-548. 10.1039/C8IB00091C PMC6193809 30116818

[DMM050352C64] de Jongh, R., Spijkers, X. M., Pasteuning-Vuhman, S., Vulto, P. and Pasterkamp, R. J. (2021). Neuromuscular junction–on–a–chip: ALS disease modeling and read–out development in microfluidic devices. *J. Neurochem.* 157, 393-412. 10.1111/jnc.15289 33382092

[DMM050352C35] Deguise, M., Baranello, G., Mastella, C., Beauvais, A., Michaud, J., Leone, A., De Amicis, R., Battezzati, A., Dunham, C., Selby, K. et al. (2019). Abnormal fatty acid metabolism is a core component of spinal muscular atrophy. *Ann. Clin. Transl. Neurol.* 6, 1519-1532. 10.1002/acn3.50855 31402618 PMC6689695

[DMM050352C36] Dimitriadi, M., Derdowski, A., Kalloo, G., Maginnis, M. S., O'Hern, P., Bliska, B., Sorkaç, A., Nguyen, K. C. Q., Cook, S. J., Poulogiannis, G. et al. (2016). Decreased function of survival motor neuron protein impairs endocytic pathways. *Proc. Natl. Acad. Sci. USA* 113, E4377-E4386. 10.1073/pnas.1600015113 27402754 PMC4968725

[DMM050352C37] Ding, Y., Wang, M., Bu, H., Li, J., Lin, X. and Xu, X. (2023). Application of an F0-based genetic assay in adult zebrafish to identify modifier genes of an inherited cardiomyopathy. *Dis. Model. Mech.* 16, dmm049427. 10.1242/dmm.049427 35481478 PMC9239171

[DMM050352C38] Doyle, J. J., Vrancx, C., Maios, C., Labarre, A., Patten, S. A. and Parker, J. A. (2020). Modulating the ER stress response attenuates neurodegeneration in a *C. elegans* model of spinal muscular atrophy. *Dis. Model. Mech.* 13, dmm.041350. 10.1242/dmm.041350 PMC7774902 33106327

[DMM050352C39] Duque, S. I., Arnold, W. D., Odermatt, P., Li, X., Porensky, P. N., Schmelzer, L., Meyer, K., Kolb, S. J., Schümperli, D., Kaspar, B. K. et al. (2015). A large animal model of spinal muscular atrophy and correction of phenotype: SMA in Pig. *Ann. Neurol.* 77, 399-414. 10.1002/ana.24332 25516063 PMC4453930

[DMM050352C40] Ebbink, B. J., Poelman, E., Aarsen, F. K., Plug, I., Régal, L., Muentjes, C., Beek, N. A. M. E., Lequin, M. H., Ploeg, A. T. and Hout, J. M. P. (2018). Classic infantile Pompe patients approaching adulthood: a cohort study on consequences for the brain. *Dev. Med. Child Neurol.* 60, 579-586. 10.1111/dmcn.13740 29573408

[DMM050352C41] Erdos, J. and Wild, C. (2022). Mid- and long-term (at least 12 months) follow-up of patients with spinal muscular atrophy (SMA) treated with nusinersen, onasemnogene abeparvovec, risdiplam or combination therapies: A systematic review of real-world study data. *Eur. J. Paediatr. Neurol.* 39, 1-10. 10.1016/j.ejpn.2022.04.006 35533607

[DMM050352C42] Eshraghi, M., McFall, E., Gibeault, S. and Kothary, R. (2016). Effect of genetic background on the phenotype of the *Smn ^2B/-^* mouse model of spinal muscular atrophy. *Hum. Mol. Genet.* 25, 4494-4506. 10.1093/hmg/ddw278 28172892 PMC5409218

[DMM050352C43] Faustino Martins, J.-M., Fischer, C., Urzi, A., Vidal, R., Kunz, S., Ruffault, P.-L., Kabuss, L., Hube, I., Gazzerro, E., Birchmeier, C. et al. (2020). Self-organizing 3D human trunk neuromuscular organoids. *Cell Stem Cell* 26, 172-186.e6. 10.1016/j.stem.2019.12.007 31956040

[DMM050352C44] Finkel, R. S., Mercuri, E., Darras, B. T., Connolly, A. M., Kuntz, N. L., Kirschner, J., Chiriboga, C. A., Saito, K., Servais, L., Tizzano, E. et al. (2017). Nusinersen versus sham control in infantile-onset spinal muscular atrophy. *N. Engl. J. Med.* 377, 1723-1732. 10.1056/NEJMoa1702752 29091570

[DMM050352C153] Fischer, U., Liu, Q. and Dreyfuss, G. (1997). The SMN-SIP1 complex has an essential role in spliceosomal snRNP biogenesis. *Cell* 90, 1023-1029. 10.1016/s0092-8674(00)80368-2 9323130

[DMM050352C45] Fletcher, E. V., Simon, C. M., Pagiazitis, J. G., Chalif, J. I., Vukojicic, A., Drobac, E., Wang, X. and Mentis, G. Z. (2017). Reduced sensory synaptic excitation impairs motor neuron function via Kv2.1 in spinal muscular atrophy. *Nat. Neurosci.* 20, 905-916. 10.1038/nn.4561 28504671 PMC5487291

[DMM050352C46] Foust, K. D., Nurre, E., Montgomery, C. L., Hernandez, A., Chan, C. M. and Kaspar, B. K. (2009). Intravascular AAV9 preferentially targets neonatal neurons and adult astrocytes. *Nat. Biotechnol.* 27, 59-65. 10.1038/nbt.1515 19098898 PMC2895694

[DMM050352C47] Franzen, J., Georgomanolis, T., Selich, A., Kuo, C.-C., Stöger, R., Brant, L., Mulabdić, M. S., Fernandez-Rebollo, E., Grezella, C., Ostrowska, A. et al. (2021). DNA methylation changes during long-term in vitro cell culture are caused by epigenetic drift. *Commun. Biol.* 4, 598. 10.1038/s42003-021-02116-y 34011964 PMC8134454

[DMM050352C48] Frugier, T., Mitchell, N. L., Tammen, I., Houweling, P. J., Arthur, D. G., Kay, G. W., Van Diggelen, O. P., Jolly, R. D. and Palmer, D. N. (2008). A new large animal model of CLN5 neuronal ceroid lipofuscinosis in Borderdale sheep is caused by a nucleotide substitution at a consensus splice site (c.571+1G >>> A) leading to excision of exon 3. *Neurobiol. Dis.* 29, 306-315. 10.1016/j.nbd.2007.09.006 17988881 PMC2249613

[DMM050352C49] Garbes, L., Heesen, L., Holker, I., Bauer, T., Schreml, J., Zimmermann, K., Thoenes, M., Walter, M., Dimos, J., Peitz, M. et al. (2013). VPA response in SMA is suppressed by the fatty acid translocase CD36. *Hum. Mol. Genet.* 22, 398-407. 10.1093/hmg/dds437 23077215

[DMM050352C50] Gombash, S. E., Cowley, C. J., Fitzgerald, J. A., Iyer, C. C., Fried, D., McGovern, V. L., Williams, K. C., Burghes, A. H. M., Christofi, F. L., Gulbransen, B. D. et al. (2015). SMN deficiency disrupts gastrointestinal and enteric nervous system function in mice. *Hum. Mol. Genet.* 24, 3847-3860. 10.1093/hmg/ddv127 25859009 PMC4542833

[DMM050352C51] Groen, E. J. N., Talbot, K. and Gillingwater, T. H. (2018a). Advances in therapy for spinal muscular atrophy: promises and challenges. *Nat. Rev. Neurol.* 14, 214-224. 10.1038/nrneurol.2018.4 29422644

[DMM050352C52] Groen, E. J. N., Perenthaler, E., Courtney, N. L., Jordan, C. Y., Shorrock, H. K., Van Der Hoorn, D., Huang, Y.-T., Murray, L. M., Viero, G. and Gillingwater, T. H. (2018b). Temporal and tissue-specific variability of SMN protein levels in mouse models of spinal muscular atrophy. *Hum. Mol. Genet.* 27, 2851-2862. 10.1093/hmg/ddy195 29790918 PMC6077828

[DMM050352C53] Hamilton, G. and Gillingwater, T. H. (2013). Spinal muscular atrophy: going beyond the motor neuron. *Trends Mol. Med.* 19, 40-50. 10.1016/j.molmed.2012.11.002 23228902

[DMM050352C54] Hauke, J., Riessland, M., Lunke, S., Eyüpoglu, I. Y., Blümcke, I., El-Osta, A., Wirth, B. and Hahnen, E. (2009). Survival motor neuron gene 2 silencing by DNA methylation correlates with spinal muscular atrophy disease severity and can be bypassed by histone deacetylase inhibition. *Hum. Mol. Genet.* 18, 304-317. 10.1093/hmg/ddn357 18971205 PMC2638778

[DMM050352C55] Heiman-Patterson, T. D., Deitch, J. S., Blankenhorn, E. P., Erwin, K. L., Perreault, M. J., Alexander, B. K., Byers, N., Toman, I. and Alexander, G. M. (2005). Background and gender effects on survival in the TgN(SOD1-G93A)1Gur mouse model of ALS. *J. Neurol. Sci.* 236, 1-7. 10.1016/j.jns.2005.02.006 16024047

[DMM050352C56] Hensel, N., Brickwedde, H., Tsaknakis, K., Grages, A., Braunschweig, L., Lüders, K. A., Lorenz, H. M., Lippross, S., Walter, L. M., Tavassol, F. et al. (2020). Altered bone development with impaired cartilage formation precedes neuromuscular symptoms in spinal muscular atrophy. *Hum. Mol. Genet.* 29, 2662-2673. 10.1093/hmg/ddaa145 32644125

[DMM050352C57] Hensel, N., Cieri, F., Santonicola, P., Tapken, I., Schüning, T., Taiana, M., Pagliari, E., Joseph, A., Fischer, S., Heidrich, N. et al. (2021). Impairment of the neurotrophic signaling hub B-Raf contributes to motoneuron degeneration in spinal muscular atrophy. *Proc. Natl. Acad. Sci. USA* 118, e2007785118. 10.1073/pnas.2007785118 33931501 PMC8106332

[DMM050352C58] Hor, J. H., Soh, E. S.-Y., Tan, L. Y., Lim, V. J. W., Santosa, M. M., Winanto, Ho, B. X., Fan, Y., Soh, B.-S. and Ng, S.-Y. (2018). Cell cycle inhibitors protect motor neurons in an organoid model of Spinal Muscular Atrophy. *Cell Death Dis.* 9, 1100. 10.1038/s41419-018-1081-0 30368521 PMC6204135

[DMM050352C59] Hosseinibarkooie, S., Peters, M., Torres-Benito, L., Rastetter, R. H., Hupperich, K., Hoffmann, A., Mendoza-Ferreira, N., Kaczmarek, A., Janzen, E., Milbradt, J. et al. (2016). The power of human protective modifiers: PLS3 and CORO1C unravel impaired endocytosis in spinal muscular atrophy and rescue SMA phenotype. *Am. J. Hum. Genet.* 99, 647-665. 10.1016/j.ajhg.2016.07.014 27499521 PMC5011078

[DMM050352C60] Hsieh-Li, H. M., Chang, J.-G., Jong, Y.-J., Wu, M.-H., Wang, N. M., Tsai, C. H. and Li, H. (2000). A mouse model for spinal muscular atrophy. *Nat. Genet.* 24, 66-70. 10.1038/71709 10615130

[DMM050352C61] Hua, Y., Vickers, T. A., Okunola, H. L., Bennett, C. F. and Krainer, A. R. (2008). Antisense masking of an hnRNP A1/A2 intronic splicing silencer corrects SMN2 splicing in transgenic mice. *Am. J. Hum. Genet.* 82, 834-848. 10.1016/j.ajhg.2008.01.014 18371932 PMC2427210

[DMM050352C62] Hua, Y., Sahashi, K., Rigo, F., Hung, G., Horev, G., Bennett, C. F. and Krainer, A. R. (2011). Peripheral SMN restoration is essential for long-term rescue of a severe spinal muscular atrophy mouse model. *Nature* 478, 123-126. 10.1038/nature10485 21979052 PMC3191865

[DMM050352C63] Hunter, G., Powis, R. A., Jones, R. A., Groen, E. J. N., Shorrock, H. K., Lane, F. M., Zheng, Y., Sherman, D. L., Brophy, P. J. and Gillingwater, T. H. (2016). Restoration of SMN in Schwann cells reverses myelination defects and improves neuromuscular function in spinal muscular atrophy. *Hum. Mol. Genet.* 25, 2853-2861. 10.1093/hmg/ddw141 27170316 PMC5181642

[DMM050352C65] Kariya, S., Obis, T., Garone, C., Akay, T., Sera, F., Iwata, S., Homma, S. and Monani, U. R. (2014). Requirement of enhanced Survival Motoneuron protein imposed during neuromuscular junction maturation. *J. Clin. Invest* 124, 785-800. 10.1172/JCI72017 24463453 PMC3904626

[DMM050352C66] Khairallah, M.-T., Astroski, J., Custer, S. K., Androphy, E. J., Franklin, C. L. and Lorson, C. L. (2017). SMN deficiency negatively impacts red pulp macrophages and spleen development in mouse models of Spinal Muscular Atrophy. *Hum. Mol. Genet.* 26, 932-941. 10.1093/hmg/ddx008 28062667 PMC6075362

[DMM050352C67] Kim, J.-K., Jha, N. N., Feng, Z., Faleiro, M. R., Chiriboga, C. A., Wei-Lapierre, L., Dirksen, R. T., Ko, C.-P. and Monani, U. R. (2020). Muscle-specific SMN reduction reveals motor neuron–independent disease in spinal muscular atrophy models. *J. Clin. Invest* 130, 1271-1287. 10.1172/JCI131989 32039917 PMC7269591

[DMM050352C68] Kobayashi, D. T., Olson, R. J., Sly, L., Swanson, C. J., Chung, B., Naryshkin, N., Narasimhan, J., Bhattacharyya, A., Mullenix, M. and Chen, K. S. (2011). Utility of survival motor neuron ELISA for spinal muscular atrophy clinical and preclinical analyses. *PLoS ONE* 6, e24269. 10.1371/journal.pone.0024269 21904622 PMC3164180

[DMM050352C69] Koch, C. M., Suschek, C. V., Lin, Q., Bork, S., Goergens, M., Joussen, S., Pallua, N., Ho, A. D., Zenke, M. and Wagner, W. (2011). Specific age-associated DNA methylation changes in human dermal fibroblasts. *PLoS ONE* 6, e16679. 10.1371/journal.pone.0016679 21347436 PMC3035656

[DMM050352C70] Ladewig, J., Mertens, J., Kesavan, J., Doerr, J., Poppe, D., Glaue, F., Herms, S., Wernet, P., Kögler, G., Müller, F.-J. et al. (2012). Small molecules enable highly efficient neuronal conversion of human fibroblasts. *Nat. Methods* 9, 575-578. 10.1038/nmeth.1972 22484851

[DMM050352C71] Lam, J., Baello, S., Iqbal, M., Kelly, L. E., Shannon, P. T., Chitayat, D., Matthews, S. G. and Koren, G. (2015). The ontogeny of P-glycoprotein in the developing human blood–brain barrier: implication for opioid toxicity in neonates. *Pediatr. Res.* 78, 417-421. 10.1038/pr.2015.119 26086643

[DMM050352C72] Lauria, F., Bernabò, P., Tebaldi, T., Groen, E. J. N., Perenthaler, E., Maniscalco, F., Rossi, A., Donzel, D., Clamer, M., Marchioretto, M. et al. (2020). SMN-primed ribosomes modulate the translation of transcripts related to spinal muscular atrophy. *Nat. Cell Biol.* 22, 1239-1251. 10.1038/s41556-020-00577-7 32958857 PMC7610479

[DMM050352C73] Le, T. T., Pham, L. T., Butchbach, M. E. R., Zhang, H. L., Monani, U. R., Coovert, D. D., Gavrilina, T. O., Xing, L., Bassell, G. J. and Burghes, A. H. M. (2005). SMNΔ7, the major product of the centromeric survival motor neuron (SMN2) gene, extends survival in mice with spinal muscular atrophy and associates with full-length SMN. *Hum. Mol. Genet.* 14, 845-857. 10.1093/hmg/ddi078 15703193

[DMM050352C74] Lefebvre, S., Bürglen, L., Reboullet, S., Clermont, O., Burlet, P., Viollet, L., Benichou, B., Cruaud, C., Millasseau, P., Zeviani, M. et al. (1995). Identification and characterization of a spinal muscular atrophy-determining gene. *Cell* 80, 155-165. 10.1016/0092-8674(95)90460-3 7813012

[DMM050352C75] Li, J., Geisbush, T. R., Arnold, W. D., Rosen, G. D., Zaworski, P. G. and Rutkove, S. B. (2014). A comparison of three electrophysiological methods for the assessment of disease status in a mild spinal muscular atrophy mouse model. *PLoS ONE* 9, e111428. 10.1371/journal.pone.0111428 25347197 PMC4210182

[DMM050352C76] Lin, T.-L., Chen, T.-H., Hsu, Y.-Y., Cheng, Y.-H., Juang, B.-T. and Jong, Y.-J. (2016). Selective neuromuscular denervation in taiwanese severe SMA mouse can be reversed by morpholino antisense oligonucleotides. *PLOS ONE* 11, e0154723. 10.1371/journal.pone.0154723 27124114 PMC4849667

[DMM050352C154] Liu, Q., Fischer, U., Wang, F. and Dreyfuss, G. (1997). The spinal muscular atrophy disease gene product, SMN, and its associated protein SIP1 are in a complex with spliceosomal snRNP proteins. *Cell* 90, 1013-1021. 10.1016/s0092-8674(00)80367-0 9323129

[DMM050352C77] Lorson, C. L., Hahnen, E., Androphy, E. J. and Wirth, B. (1999). A single nucleotide in the SMN gene regulates splicing and is responsible for spinal muscular atrophy. *Proc. Natl Acad. Sci. USA* 96, 6307-6311. 10.1073/pnas.96.11.6307 10339583 PMC26877

[DMM050352C78] Lotti, F., Imlach, W. L., Saieva, L., Beck, E. S., Hao, L. T., Li, D. K., Jiao, W., Mentis, G. Z., Beattie, C. E., McCabe, B. D. et al. (2012). An SMN-dependent U12 splicing event essential for motor circuit function. *Cell* 151, 440-454. 10.1016/j.cell.2012.09.012 23063131 PMC3474596

[DMM050352C79] Lutz, C. (2018). Mouse models of ALS: Past, present and future. *Brain Res.* 1693, 1-10. 10.1016/j.brainres.2018.03.024 29577886

[DMM050352C80] Lutz, C. M., Kariya, S., Patruni, S., Osborne, M. A., Liu, D., Henderson, C. E., Li, D. K., Pellizzoni, L., Rojas, J., Valenzuela, D. M. et al. (2011). Postsymptomatic restoration of SMN rescues the disease phenotype in a mouse model of severe spinal muscular atrophy. *J. Clin. Invest.* 121, 3029-3041. 10.1172/JCI57291 21785219 PMC3148744

[DMM050352C81] McWhorter, M. L., Monani, U. R., Burghes, A. H. M. and Beattie, C. E. (2003). Knockdown of the survival motor neuron (Smn) protein in zebrafish causes defects in motor axon outgrowth and pathfinding. *J. Cell Biol.* 162, 919-932. 10.1083/jcb.200303168 12952942 PMC1761110

[DMM050352C82] Mendell, J. R., Al-Zaidy, S., Shell, R., Arnold, W. D., Rodino-Klapac, L. R., Prior, T. W., Lowes, L., Alfano, L., Berry, K., Church, K. et al. (2017). Single-dose gene-replacement therapy for spinal muscular atrophy. *N. Engl. J. Med.* 377, 1713-1722. 10.1056/NEJMoa1706198 29091557

[DMM050352C83] Mercuri, E., Darras, B. T., Chiriboga, C. A., Day, J. W., Campbell, C., Connolly, A. M., Iannaccone, S. T., Kirschner, J., Kuntz, N. L., Saito, K. et al. (2018). Nusinersen versus sham control in later-onset spinal muscular atrophy. *N. Engl. J. Med.* 378, 625-635. 10.1056/NEJMoa1710504 29443664

[DMM050352C84] Mercuri, E., Sumner, C. J., Muntoni, F., Darras, B. T. and Finkel, R. S. (2022). Spinal muscular atrophy. *Nat. Rev. Dis. Primer* 8, 1-16. 10.1038/s41572-022-00380-8 35927425

[DMM050352C85] Michaud, M., Arnoux, T., Bielli, S., Durand, E., Rotrou, Y., Jablonka, S., Robert, F., Giraudon-Paoli, M., Riessland, M., Mattei, M.-G. et al. (2010). Neuromuscular defects and breathing disorders in a new mouse model of spinal muscular atrophy. *Neurobiol. Dis.* 38, 125-135. 10.1016/j.nbd.2010.01.006 20085811

[DMM050352C86] Monani, U. R., Lorson, C. L., Parsons, D. W., Prior, T. W., Androphy, E. J., Burghes, A. H. M. and McPherson, J. D. (1999). A single nucleotide difference that alters splicing patterns distinguishes the SMA gene SMN1 from the copy gene SMN2. *Hum. Mol. Genet.* 8, 1177-1183. 10.1093/hmg/8.7.1177 10369862

[DMM050352C87] Monani, U. R., Sendtner, M., Coovert, D. D., Parsons, D. W., Andreassi, C., Le, T. T., Jablonka, S., Schrank, B., Rossol, W., Prior, T. W. et al. (2000). The human centromeric survival motor neuron gene (SMN2) rescues embryonic lethality in Smn–/– mice and results in a mouse with spinal muscular atrophy. *Hum. Mol. Genet.* 9, 333-339. 10.1093/hmg/9.3.333 10655541

[DMM050352C88] Monani, U. R., Pastore, M. T., Gavrilina, T. O., Jablonka, S., Le, T. T., Andreassi, C., DiCocco, J. M., Lorson, C., Androphy, E. J., Sendtner, M. et al. (2003). A transgene carrying an A2G missense mutation in the SMN gene modulates phenotypic severity in mice with severe (type I) spinal muscular atrophy. *J. Cell Biol.* 160, 41-52. 10.1083/jcb.200208079 12515823 PMC2172739

[DMM050352C89] Murray, L. M., Comley, L. H., Thomson, D., Parkinson, N., Talbot, K. and Gillingwater, T. H. (2008). Selective vulnerability of motor neurons and dissociation of pre- and post-synaptic pathology at the neuromuscular junction in mouse models of spinal muscular atrophy. *Hum. Mol. Genet.* 17, 949-962. 10.1093/hmg/ddm367 18065780

[DMM050352C90] Naryshkin, N. A., Weetall, M., Dakka, A., Narasimhan, J., Zhao, X., Feng, Z., Ling, K. K. Y., Karp, G. M., Qi, H., Woll, M. G. et al. (2014). *SMN2* splicing modifiers improve motor function and longevity in mice with spinal muscular atrophy. *Science* 345, 688-693. 10.1126/science.1250127 25104390

[DMM050352C91] Nguyen, V. V. T., Ye, S., Gkouzioti, V., Van Wolferen, M. E., Yengej, F. Y., Melkert, D., Siti, S., De Jong, B., Besseling, P. J., Spee, B. et al. (2022). A human kidney and liver organoid–based multi–organ–on–a–chip model to study the therapeutic effects and biodistribution of mesenchymal stromal cell–derived extracellular vesicles. *J. Extracell. Vesicles* 11, 12280. 10.1002/jev2.12280 36382606 PMC9667402

[DMM050352C92] Nicole, S., Desforges, B., Millet, G., Lesbordes, J., Cifuentes-Diaz, C., Vertes, D., Cao, M. L., De Backer, F., Languille, L., Roblot, N. et al. (2003). Intact satellite cells lead to remarkable protection against *Smn* gene defect in differentiated skeletal muscle. *J. Cell Biol.* 161, 571-582. 10.1083/jcb.200210117 12743106 PMC2172949

[DMM050352C93] Nölle, A., Zeug, A., Van Bergeijk, J., Tönges, L., Gerhard, R., Brinkmann, H., Al Rayes, S., Hensel, N., Schill, Y., Apkhazava, D. et al. (2011). The spinal muscular atrophy disease protein SMN is linked to the rho-kinase pathway via profilin. *Hum. Mol. Genet.* 20, 4865-4878. 10.1093/hmg/ddr425 21920940

[DMM050352C94] O'Hern, P., Garcia, E. L., Hao, L. T., Hart, A. C., Matera, A. G. and Beattie, C. E. (2017). Nonmammalian animal models of spinal muscular atrophy. In *Spinal muscular atrophy - Disease mechanisms and* therapy (ed. C. J. Sumner, S. Paushkin and C.-P. Ko), pp. 221-239. Academic Press. 10.1016/B978-0-12-803685-3.00014-8

[DMM050352C95] Oprea, G. E., Kröber, S., McWhorter, M. L., Rossoll, W., Müller, S., Krawczak, M., Bassell, G. J., Beattie, C. E. and Wirth, B. (2008). Plastin 3 is a protective modifier of autosomal recessive spinal muscular atrophy. *Science* 320, 524-527. 10.1126/science.1155085 18440926 PMC4908855

[DMM050352C96] Oprişoreanu, A.-M., Smith, H. L., Krix, S., Chaytow, H., Carragher, N. O., Gillingwater, T. H., Becker, C. G. and Becker, T. (2021). Automated *in vivo* drug screen in zebrafish identifies synapse-stabilising drugs with relevance to spinal muscular atrophy. *Dis. Model. Mech.* 14, dmm047761. 10.1242/dmm.047761 33973627 PMC8106959

[DMM050352C97] Osborne, M., Gomez, D., Feng, Z., McEwen, C., Beltran, J., Cirillo, K., El-Khodor, B., Lin, M.-Y., Li, Y., Knowlton, W. M. et al. (2012). Characterization of behavioral and neuromuscular junction phenotypes in a novel allelic series of SMA mouse models. *Hum. Mol. Genet.* 21, 4431-4447. 10.1093/hmg/dds285 22802075 PMC3459466

[DMM050352C98] Osman, E. Y., Bolding, M. R., Villalón, E., Kaifer, K. A., Lorson, Z. C., Tisdale, S., Hao, Y., Conant, G. C., Pires, J. C., Pellizzoni, L. et al. (2019). Functional characterization of SMN evolution in mouse models of SMA. *Sci. Rep.* 9, 9472. 10.1038/s41598-019-45822-8 31263170 PMC6603021

[DMM050352C99] Palladino, A., Passamano, L., Taglia, A., D'Ambrosio, P., Scutifero, M., Cecio, M. R., Picillo, E., Viggiano, E., Torre, V., De Luca, F. et al. (2011). Cardiac involvement in patients with spinal muscular atrophies. *Acta Myol.* 30, 175-178. 22616198 PMC3298107

[DMM050352C100] Park, G.-H., Maeno-Hikichi, Y., Awano, T., Landmesser, L. T. and Monani, U. R. (2010). Reduced Survival of Motor Neuron (SMN) protein in motor neuronal progenitors functions cell autonomously to cause spinal muscular atrophy in model mice expressing the human centromeric (SMN2) gene. *J. Neurosci.* 30, 12005-12019. 10.1523/JNEUROSCI.2208-10.2010 20826664 PMC2944776

[DMM050352C101] Piepers, S., Berg, L. H., Brugman, F., Scheffer, H., Ruiterkamp-Versteeg, M., Engelen, B. G., Faber, C. G., Visser, M., Pol, W.-L. and Wokke, J. H. J. (2008). A natural history study of late onset spinal muscular atrophy types 3b and 4. *J. Neurol.* 255, 1400-1404. 10.1007/s00415-008-0929-0 18575920

[DMM050352C102] Poirier, A., Weetall, M., Heinig, K., Bucheli, F., Schoenlein, K., Alsenz, J., Bassett, S., Ullah, M., Senn, C., Ratni, H. et al. (2018). Risdiplam distributes and increases SMN protein in both the central nervous system and peripheral organs. *Pharmacol. Res. Perspect* 6, e00447. 10.1002/prp2.447 30519476 PMC6262736

[DMM050352C103] Powis, R. A., Karyka, E., Boyd, P., Côme, J., Jones, R. A., Zheng, Y., Szunyogova, E., Groen, E. J. N., Hunter, G., Thomson, D. et al. (2016). Systemic restoration of UBA1 ameliorates disease in spinal muscular atrophy. *JCI Insight* 1, e87908. 10.1172/jci.insight.87908 27699224 PMC5033939

[DMM050352C104] Ramos, D. M., d'Ydewalle, C., Gabbeta, V., Dakka, A., Klein, S. K., Norris, D. A., Matson, J., Taylor, S. J., Zaworski, P. G., Prior, T. W. et al. (2019). Age-dependent SMN expression in disease-relevant tissue and implications for SMA treatment. *J. Clin. Invest* 129, 4817-4831. 10.1172/JCI124120 31589162 PMC6819103

[DMM050352C105] Ribitsch, I., Baptista, P. M., Lange-Consiglio, A., Melotti, L., Patruno, M., Jenner, F., Schnabl-Feichter, E., Dutton, L. C., Connolly, D. J., Van Steenbeek, F. G. et al. (2020). Large animal models in regenerative medicine and tissue engineering: to do or not to do. *Front. Bioeng. Biotechnol.* 8, 972. 10.3389/fbioe.2020.00972 32903631 PMC7438731

[DMM050352C106] Riessland, M., Kaczmarek, A., Schneider, S., Swoboda, K. J., Löhr, H., Bradler, C., Grysko, V., Dimitriadi, M., Hosseinibarkooie, S., Torres-Benito, L. et al. (2017). Neurocalcin delta suppression protects against spinal muscular atrophy in humans and across species by restoring impaired endocytosis. *Am. J. Hum. Genet.* 100, 297-315. 10.1016/j.ajhg.2017.01.005 28132687 PMC5294679

[DMM050352C107] Rochette, C., Gilbert, N. and Simard, L. (2001). SMN gene duplication and the emergence of the SMN2 gene occurred in distinct hominids: SMN2 is unique to Homo sapiens. *Hum. Genet.* 108, 255-266. 10.1007/s004390100473 11354640

[DMM050352C108] Rudnik-Schöneborn, S., Heller, R., Berg, C., Betzler, C., Grimm, T., Eggermann, T., Eggermann, K., Wirth, R., Wirth, B. and Zerres, K. (2008). Congenital heart disease is a feature of severe infantile spinal muscular atrophy. *J. Med. Genet.* 45, 635-638. 10.1136/jmg.2008.057950 18662980

[DMM050352C109] Ruiz, R. and Tabares, L. (2014). Neurotransmitter release in motor nerve terminals of a mouse model of mild spinal muscular atrophy. *J. Anat.* 224, 74-84. 10.1111/joa.12038 23489475 PMC3867889

[DMM050352C110] Sahashi, K., Ling, K. K. Y., Hua, Y., Wilkinson, J. E., Nomakuchi, T., Rigo, F., Hung, G., Xu, D., Jiang, Y., Lin, R. Z. et al. (2013). Pathological impact of *SMN 2* mis–splicing in adult SMA mice. *EMBO Mol. Med.* 5, 1586-1601. 10.1002/emmm.201302567 24014320 PMC3799581

[DMM050352C111] Scheijmans, F. E. V., Cuppen, I., Van Eijk, R. P. A., Wijngaarde, C. A., Schoenmakers, M. A. G. C., Van Der Woude, D. R., Bartels, B., Veldhoen, E. S., Oude Lansink, I. L. B., Groen, E. J. N. et al. (2022). Population-based assessment of nusinersen efficacy in children with spinal muscular atrophy: a 3-year follow-up study. *Brain Commun.* 4, fcac269. 10.1093/braincomms/fcac269 36382221 PMC9651026

[DMM050352C112] Schelstraete, W., Clerck, L. D., Govaert, E., Millecam, J., Devreese, M., Deforce, D., Bocxlaer, J. V. and Croubels, S. (2019). Characterization of porcine hepatic and intestinal drug metabolizing CYP450: comparison with human orthologues from a quantitative, activity and selectivity perspective. *Sci. Rep.* 9, 9233. 10.1038/s41598-019-45212-0 31239454 PMC6592956

[DMM050352C113] Schrank, B., Götz, R., Gunnersen, J. M., Ure, J. M., Toyka, K. V., Smith, A. G. and Sendtner, M. (1997). Inactivation of the survival motor neuron gene, a candidate gene for human spinal muscular atrophy, leads to massive cell death in early mouse embryos. *Proc. Natl. Acad. Sci. USA* 94, 9920-9925. 10.1073/pnas.94.18.9920 9275227 PMC23295

[DMM050352C114] Schreml, J., Riessland, M., Paterno, M., Garbes, L., Roßbach, K., Ackermann, B., Krämer, J., Somers, E., Parson, S. H., Heller, R. et al. (2013). Severe SMA mice show organ impairment that cannot be rescued by therapy with the HDACi JNJ-26481585. *Eur. J. Hum. Genet.* 21, 643-652. 10.1038/ejhg.2012.222 23073311 PMC3658191

[DMM050352C115] Shababi, M., Habibi, J., Yang, H. T., Vale, S. M., Sewell, W. A. and Lorson, C. L. (2010). Cardiac defects contribute to the pathology of spinal muscular atrophy models. *Hum. Mol. Genet.* 19, 4059-4071. 10.1093/hmg/ddq329 20696672

[DMM050352C116] Shababi, M., Habibi, J., Ma, L., Glascock, J. J., Sowers, J. R. and Lorson, C. L. (2012). Partial restoration of cardio-vascular defects in a rescued severe model of spinal muscular atrophy. *J. Mol. Cell. Cardiol.* 52, 1074-1082. 10.1016/j.yjmcc.2012.01.005 22285962 PMC3327806

[DMM050352C117] Shanmugarajan, S., Tsuruga, E., Swoboda, K. J., Maria, B. L., Ries, W. L. and Reddy, S. V. (2009). Bone loss in survival motor neuron (*Smn* ^−/−^ *SMN2*) genetic mouse model of spinal muscular atrophy: Bone loss in a mouse model of spinal muscular atrophy. *J. Pathol.* 219, 52-60. 10.1002/path.2566 19434631 PMC2791336

[DMM050352C118] Sharifi, Z., Taheri, M., Fallah, M.-S., Abiri, M., Golnabi, F., Bagherian, H., Zeinali, R., Farahzadi, H., Alborji, M., Tehrani, P. G. et al. (2021). Comprehensive mutation analysis and report of 12 novel mutations in a cohort of patients with spinal muscular atrophy in Iran. *J. Mol. Neurosci.* 71, 2281-2298. 10.1007/s12031-020-01789-0 33481221

[DMM050352C119] Singh, N. K., Singh, N. N., Androphy, E. J. and Singh, R. N. (2006). Splicing of a critical exon of human *survival motor neuron* is regulated by a unique silencer element located in the last intron. *Mol. Cell. Biol.* 26, 1333-1346. 10.1128/MCB.26.4.1333-1346.2006 16449646 PMC1367187

[DMM050352C120] Sintusek, P., Catapano, F., Angkathunkayul, N., Marrosu, E., Parson, S. H., Morgan, J. E., Muntoni, F. and Zhou, H. (2016). Histopathological defects in intestine in severe spinal muscular atrophy mice are improved by systemic antisense oligonucleotide treatment. *PLOS ONE* 11, e0155032. 10.1371/journal.pone.0155032 27163330 PMC4862622

[DMM050352C121] Sleigh, J. N., Buckingham, S. D., Esmaeili, B., Viswanathan, M., Cuppen, E., Westlund, B. M. and Sattelle, D. B. (2011a). A novel Caenorhabditis elegans allele, smn-1(cb131), mimicking a mild form of spinal muscular atrophy, provides a convenient drug screening platform highlighting new and pre-approved compounds. *Hum. Mol. Genet.* 20, 245-260. 10.1093/hmg/ddq459 20962036

[DMM050352C122] Sleigh, J. N., Gillingwater, T. H. and Talbot, K. (2011b). The contribution of mouse models to understanding the pathogenesis of spinal muscular atrophy. *Dis. Model. Mech.* 4, 457-467. 10.1242/dmm.007245 21708901 PMC3124050

[DMM050352C123] Šoltić, D., Shorrock, H. K., Allardyce, H., Wilson, E. L., Holt, I., Synowsky, S. A., Shirran, S. L., Parson, S. H., Gillingwater, T. H. and Fuller, H. R. (2019). Lamin A/C dysregulation contributes to cardiac pathology in a mouse model of severe spinal muscular atrophy. *Hum. Mol. Genet.* 28, 3515-3527. 10.1093/hmg/ddz195 31397869 PMC6927462

[DMM050352C124] Somers, E., Lees, R. D., Hoban, K., Sleigh, J. N., Zhou, H., Muntoni, F., Talbot, K., Gillingwater, T. H. and Parson, S. H. (2016). Vascular defects and spinal cord hypoxia in spinal muscular atrophy: depleted vasculature and hypoxia in SMA. *Ann. Neurol.* 79, 217-230. 10.1002/ana.24549 26506088

[DMM050352C125] Southam, K. A., King, A. E., Blizzard, C. A., McCormack, G. H. and Dickson, T. C. (2013). Microfluidic primary culture model of the lower motor neuron–neuromuscular junction circuit. *J. Neurosci. Methods* 218, 164-169. 10.1016/j.jneumeth.2013.06.002 23774648

[DMM050352C126] Spring, A. M., Raimer, A. C., Hamilton, C. D., Schillinger, M. J. and Matera, A. G. (2019). Comprehensive Modeling of Spinal Muscular Atrophy in Drosophila melanogaster. *Front. Mol. Neurosci.* 12, 113. 10.3389/fnmol.2019.00113 31156382 PMC6532329

[DMM050352C127] Strauss, K. A., Farrar, M. A., Muntoni, F., Saito, K., Mendell, J. R., Servais, L., McMillan, H. J., Finkel, R. S., Swoboda, K. J., Kwon, J. M. et al. (2022a). Onasemnogene abeparvovec for presymptomatic infants with two copies of SMN2 at risk for spinal muscular atrophy type 1: the Phase III SPR1NT trial. *Nat. Med.* 28, 1381-1389. 10.1038/s41591-022-01866-4 35715566 PMC9205281

[DMM050352C128] Strauss, K. A., Farrar, M. A., Muntoni, F., Saito, K., Mendell, J. R., Servais, L., McMillan, H. J., Finkel, R. S., Swoboda, K. J., Kwon, J. M. et al. (2022b). Onasemnogene abeparvovec for presymptomatic infants with three copies of SMN2 at risk for spinal muscular atrophy: the Phase III SPR1NT trial. *Nat. Med.* 28, 1390-1397. 10.1038/s41591-022-01867-3 35715567 PMC9205287

[DMM050352C129] Takahashi, K. and Yamanaka S. (2006) Induction of pluripotent stem cells from mouse embryonic and adult fibroblast cultures by defined factors. *Cell* 126, 663-676. 10.1016/j.cell.2006.07.024 16904174

[DMM050352C130] Tay, S. H., Ellieyana, E. N., Le, Y., Sarusie, M. V., Grimm, C., Ohmer, J., Mathuru, A. S., Fischer, U. and Winkler, C. (2021). A novel zebrafish model for intermediate type spinal muscular atrophy demonstrates importance of Smn for maintenance of mature motor neurons. *Hum. Mol. Genet.* 30, 2488-2502. 10.1093/hmg/ddab212 34302176

[DMM050352C131] Thomsen, G., Burghes, A. H. M., Hsieh, C., Do, J., Chu, B. T. T., Perry, S., Barkho, B., Kaufmann, P., Sproule, D. M., Feltner, D. E. et al. (2021). Biodistribution of onasemnogene abeparvovec DNA, mRNA and SMN protein in human tissue. *Nat. Med.* 27, 1701-1711. 10.1038/s41591-021-01483-7 34608334

[DMM050352C132] Thomson, A. K., Somers, E., Powis, R. A., Shorrock, H. K., Murphy, K., Swoboda, K. J., Gillingwater, T. H. and Parson, S. H. (2017). Survival of motor neurone protein is required for normal postnatal development of the spleen. *J. Anat.* 230, 337-346. 10.1111/joa.12546 27726134 PMC5244459

[DMM050352C133] Valori, C. F., Ning, K., Wyles, M., Mead, R. J., Grierson, A. J., Shaw, P. J. and Azzouz, M. (2010). Systemic delivery of scAAV9 expressing SMN prolongs survival in a model of spinal muscular atrophy. *Sci. Transl. Med.* 2, 35ra42. 10.1126/scitranslmed.3000830 20538619

[DMM050352C134] Varderidou-Minasian, S., Verheijen, B. M., Harschnitz, O., Kling, S., Karst, H., Van Der Pol, W. L., Pasterkamp, R. J. and Altelaar, M. (2021). Spinal muscular atrophy patient iPSC-derived motor neurons display altered proteomes at early stages of differentiation. *ACS Omega* 6, 35375-35388. 10.1021/acsomega.1c04688 34984269 PMC8717385

[DMM050352C135] Vitte, J. M., Davoult, B., Roblot, N., Mayer, M., Joshi, V., Courageot, S., Tronche, F., Vadrot, J., Moreau, M. H., Kemeny, F. et al. (2004). Deletion of murine Smn exon 7 directed to liver leads to severe defect of liver development associated with iron overload. *Am. J. Pathol.* 165, 1731-1741. 10.1016/S0002-9440(10)63428-1 15509541 PMC1618680

[DMM050352C136] Wadman, R. I., Stam, M., Jansen, M. D., Van Der Weegen, Y., Wijngaarde, C. A., Harschnitz, O., Sodaar, P., Braun, K. P. J., Dooijes, D., Lemmink, H. H. et al. (2016). A comparative study of SMN protein and mRNA in blood and fibroblasts in patients with spinal muscular atrophy and healthy controls. *PLOS ONE* 11, e0167087. 10.1371/journal.pone.0167087 27893852 PMC5125671

[DMM050352C137] Wadman, R. I., Stam, M., Gijzen, M., Lemmink, H. H., Snoeck, I. N., Wijngaarde, C. A., Braun, K. P. J., Schoenmakers, M. A. G. C., Van Den Berg, L. H., Dooijes, D. et al. (2017). Association of motor milestones, SMN2 copy and outcome in spinal muscular atrophy types 0–4. *J. Neurol. Neurosurg. Psychiatry* 88, 365-367. 10.1136/jnnp-2016-314292 28108522

[DMM050352C138] Wadman, R. I., Jansen, M. D., Stam, M., Wijngaarde, C. A., Curial, C. A. D., Medic, J., Sodaar, P., Schouten, J., Vijzelaar, R., Lemmink, H. H. et al. (2020). Intragenic and structural variation in the SMN locus and clinical variability in spinal muscular atrophy. *Brain Commun.* 2, fcaa075. 10.1093/braincomms/fcaa075 32954327 PMC7425299

[DMM050352C139] Wan, B., Feng, P., Guan, Z., Sheng, L., Liu, Z. and Hua, Y. (2018). A severe mouse model of spinal muscular atrophy develops early systemic inflammation. *Hum. Mol. Genet.* 27, 4061-4076. 10.1093/hmg/ddy300 30137324

[DMM050352C155] Wang, J., Schultz, P. G. and Johnson, K. A. (2018). Mechanistic studies of a small-molecule modulator of SMN2 splicing. *Proc. Natl Acad. Sci. USA.* 115, E4604-E4612. 10.1073/pnas.1800260115 29712837 PMC5960314

[DMM050352C140] Weissman, A. S., Kennedy, K. R., Powell, M. R. and Davis, L. S. (2021). Skin necrosis in spinal muscular atrophy: Case report and review of the literature. *Pediatr. Dermatol.* 38, 632-636. 10.1111/pde.14538 33619801

[DMM050352C141] Wijngaarde, C. A., Blank, A. C., Stam, M., Wadman, R. I., van den Berg, L. H. and van der Pol, W. L. (2017). Cardiac pathology in spinal muscular atrophy: a systematic review. *Orphanet J. Rare Dis.* 12, 67. 10.1186/s13023-017-0613-5 28399889 PMC5387385

[DMM050352C142] Wijngaarde, C. A., Stam, M., Otto, L. A. M., Van Eijk, R. P. A., Cuppen, I., Veldhoen, E. S., Van Den Berg, L. H., Wadman, R. I. and Van Der Pol, W. L. (2020). Population-based analysis of survival in spinal muscular atrophy. *Neurology* 94, e1634-e1644. 10.1212/WNL.0000000000009248 32217777

[DMM050352C143] Wirth, B., Karakaya, M., Kye, M. J. and Mendoza-Ferreira, N. (2020). Twenty-five years of spinal muscular atrophy research: from phenotype to genotype to therapy, and what comes next. *Annu. Rev. Genomics Hum. Genet.* 21, 231-261. 10.1146/annurev-genom-102319-103602 32004094

[DMM050352C144] Wishart, T. M., Mutsaers, C. A., Riessland, M., Reimer, M. M., Hunter, G., Hannam, M. L., Eaton, S. L., Fuller, H. R., Roche, S. L., Somers, E. et al. (2014). Dysregulation of ubiquitin homeostasis and β-catenin signaling promote spinal muscular atrophy. *J. Clin. Invest* 124, 1821-1834. 10.1172/JCI71318 24590288 PMC3973095

[DMM050352C145] Wohnrade, C., Velling, A.-K., Mix, L., Wurster, C. D., Cordts, I., Stolte, B., Zeller, D., Uzelac, Z., Platen, S., Hagenacker, T. et al. (2023). Health-related quality of life in spinal muscular atrophy patients and their caregivers—a prospective, cross-sectional, multi-center analysis. *Brain Sci.* 13, 110. 10.3390/brainsci13010110 36672091 PMC9857112

[DMM050352C146] Workman, E., Saieva, L., Carrel, T. L., Crawford, T. O., Liu, D., Lutz, C., Beattie, C. E., Pellizzoni, L. and Burghes, A. H. M. (2009). A SMN missense mutation complements SMN2 restoring snRNPs and rescuing SMA mice. *Hum. Mol. Genet.* 18, 2215-2229. 10.1093/hmg/ddp157 19329542 PMC2685758

[DMM050352C147] Yeo, C. J. J. and Darras, B. T. (2020). Overturning the Paradigm of spinal muscular atrophy as just a motor neuron disease. *Pediatr. Neurol.* 109, 12-19. 10.1016/j.pediatrneurol.2020.01.003 32409122

[DMM050352C148] Zaworski, P., Von Herrmann, K. M., Taylor, S., Sunshine, S. S., McCarthy, K., Risher, N., Newcomb, T., Weetall, M., Prior, T. W., Swoboda, K. J. et al. (2016). SMN protein can be reliably measured in whole blood with an electrochemiluminescence (ECL) immunoassay: implications for clinical trials. *PLOS ONE* 11, e0150640. 10.1371/journal.pone.0150640 26953792 PMC4783032

[DMM050352C149] Zhao, X., Feng, Z., Risher, N., Mollin, A., Sheedy, J., Ling, K. K. Y., Narasimhan, J., Dakka, A., Baird, J. D., Ratni, H. et al. (2021). SMN protein is required throughout life to prevent spinal muscular atrophy disease progression. *Hum. Mol. Genet.* 31, 82-96. 10.1093/hmg/ddab220 34368854

[DMM050352C150] Zhou, H., Meng, J., Marrosu, E., Janghra, N., Morgan, J. and Muntoni, F. (2015). Repeated low doses of morpholino antisense oligomer: an intermediate mouse model of spinal muscular atrophy to explore the window of therapeutic response. *Hum. Mol. Genet.* 24, 6265-6277. 10.1093/hmg/ddv329 26264577 PMC4614699

